# DKK1 Suppresses Hippo Signaling via PIP_3_–OGT–LRP6 *O*-GlcNAcylation in Hepatocellular Carcinoma

**DOI:** 10.34133/cancomm.0022

**Published:** 2026-04-27

**Authors:** Ukjin Lee, Jung Woo Eun, Taehee Kim, Hyewon Shim, Yunho Choi, Hyeryeon Jung, Jaewoo Mo, Soon Sun Kim, Geum Ok Baek, Moon Gyeong Yoon, Hyeyoon Lee, Wonhwa Lee, Wantae Kim, Junil Kim, Eugene C. Yi, Seung Up Kim, Jae Youn Cheong, Eek-Hoon Jho

**Affiliations:** ^1^ Department of Life Science, University of Seoul, Seoul 02504, Republic of Korea.; ^2^ Department of Gastroenterology, Ajou University School of Medicine, Suwon 16499, Republic of Korea.; ^3^ Department of Molecular Medicine and Biopharmaceutical Sciences, Seoul National University, Seoul 03080, Republic of Korea.; ^4^ Department of Bioinformatics, Soongsil University, Seoul 06978, Republic of Korea.; ^5^ Division of Molecular Embryology, Center for Molecular Biology of Heidelberg University–German Cancer Research Center Alliance, Heidelberg 69120, Germany.; ^6^ Department of Chemistry, Sungkyunkwan University, Suwon 16419, Republic of Korea.; ^7^ School of Systems Biomedical Science, Soongsil University, Seoul 06978, Republic of Korea.; ^8^ Department of Internal Medicine, Yonsei University College of Medicine, Seoul 03722, Republic of Korea.

Dickkopf-1 (DKK1), a canonical Wnt signaling antagonist generally regarded as a tumor suppressor, is paradoxically overexpressed in hepatocellular carcinoma (HCC) and has been linked to tumor progression [1,2]. HCC development is also driven by suppression of Hippo signaling and yes-associated protein (YAP) activation [3]. However, whether elevated DKK1 functionally relates to Hippo suppression in HCC remains unclear.

Analyses of The Cancer Genome Atlas (TCGA) showed that *DKK1* mRNA levels were consistently elevated in HCC harboring tumor protein P53 (*TP53*), catenin beta 1 (*CTNNB1*), or axis inhibition protein 1 (*AXIN1*) mutations compared to normal tissues. In addition, transcriptome analyses revealed significant enrichment of TEA domain transcription factor (TEAD) 2 and 4 in *DKK1*-high tumors relative to *DKK1*-negative tumors (Fig. S1A and B). Single-cell RNA sequencing of a public HCC dataset (GSE149614) similarly showed that tumor hepatocytes exhibit elevated *DKK1* expression alongside up-regulation of YAP targets *CTGF* and *CYR61* (Fig. S1C to G) [4]. Immunohistochemical analysis (IHC) of 67 HCC samples revealed increased DKK1, YAP, and CTGF protein levels versus normal liver samples, with positive correlations among their protein levels (Fig. 1A and Fig. S2A). Because DKK1 is highly expressed during early-stage HCC and has been proposed as an early diagnostic marker [2], we hypothesized that it could potentially drive Hippo pathway suppression and YAP activation. To test this, a xenograft mouse model was generated using Hep3B cells, as these cells exhibited the highest level of DKK1 expression (Fig. S2B to D). Oral administration of the DKK1 inhibitor WAY-262611 markedly reduced YAP and YAP target gene expression and decreased tumor size and weight, demonstrating a potent antitumor effect in vivo (Fig. 1B and Fig. S2E to G). Consistently, DKK1 knockdown led to the down-regulation of YAP target genes, whereas DKK1-conditioned medium (CM) treatment induced YAP target gene expression in HCC cell lines (Fig. 1C and Fig. S2H and I). DKK1-CM promoted migration, invasion, and wound healing in Huh7 cells, but these effects were abolished by YAP/TAZ double knockdown or TEAD inhibition (MGH-CP1), indicating that DKK1-induced progression is YAP/TAZ-dependent (Fig. S3). A proximity ligation assay (PLA) further revealed a reduced TEAD1–YAP interaction upon DKK1 knockdown (Fig. S4A). Although *YAP* and *TAZ* mRNA levels remained unchanged under DKK1 knockdown or DKK1-CM treatment, YAP protein levels were markedly reduced in DKK1-knockdown and DKK1-knockout (KO) cells (Fig. S4B to F). Moreover, recombinant human DKK1 (rhDKK1) or DKK1-CM treatment promoted nuclear localization of YAP, whereas transient DKK1 silencing caused cytosolic retention (Fig. 1D and Fig. S4G and H). DKK1 treatment reduced YAP phosphorylation and large tumor suppressor kinase 1 (LATS1) phosphorylation without altering mammalian STE20-like protein kinase 1 (MST1) phosphorylation (Fig. S4I to K), while DKK1 knockdown increased YAP and LATS1 phosphorylation (Fig. 1E and Fig. S4J). Because moesin–ezrin–radixin-like protein (Merlin)–LATS1/2 interaction facilitates LATS1/2 recruitment to the plasma membrane for MST1-mediated activation [5], we assessed membrane-associated LATS1. DKK1-CM or rhDKK1 reduced the membrane pool of LATS1 and weakened LATS1–Merlin interaction (Fig. S5A to C), whereas WAY-262611 enhanced this interaction (Fig. S5D). Collectively, the above findings show that DKK1 could drive YAP-dependent HCC progression by disrupting Merlin–LATS1 interaction and suppressing Hippo signaling.

**Fig. 1.**
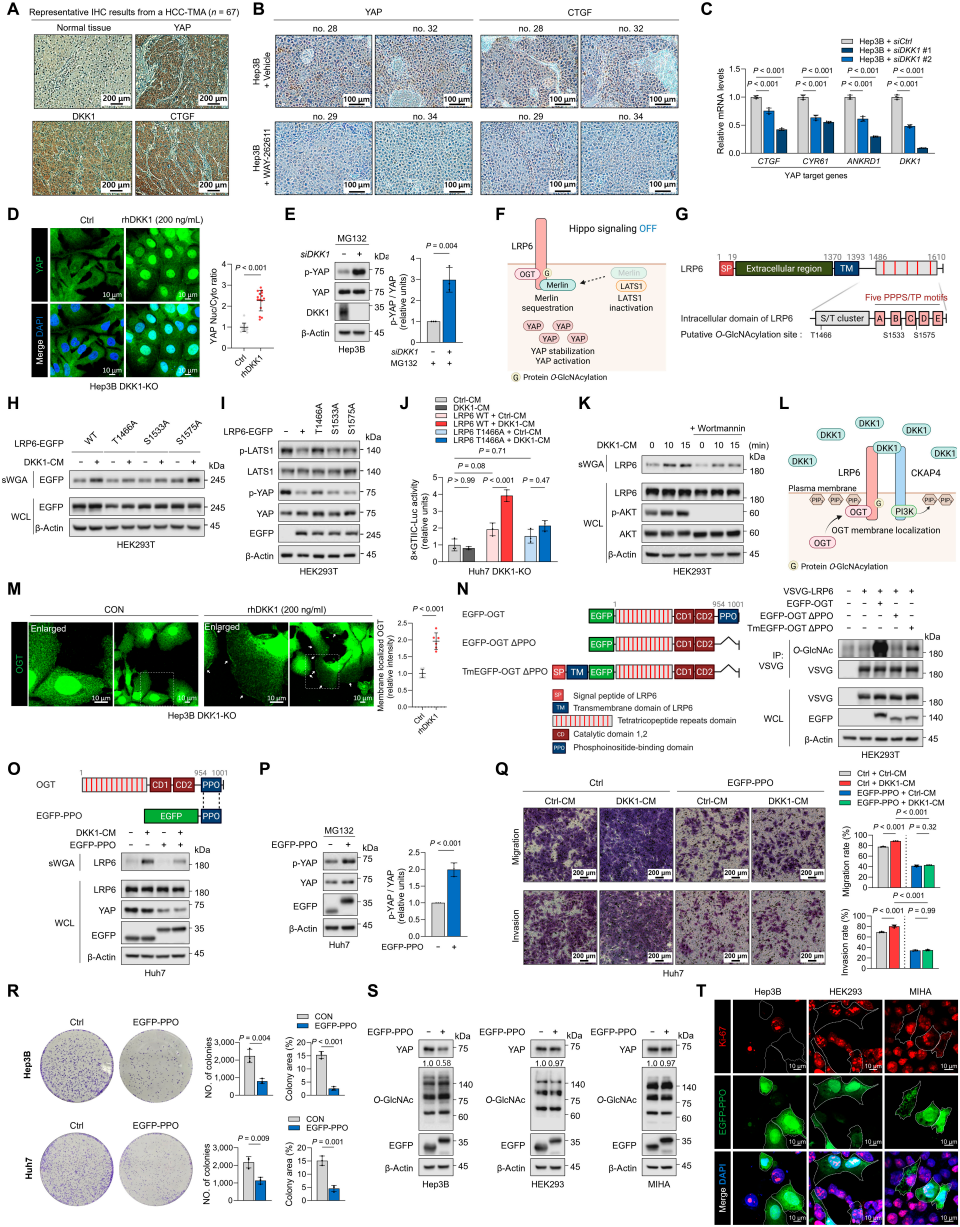
DKK1 activates the PIP_3_–OGT–LRP6 axis to suppress Hippo signaling in hepatocellular carcinoma. (A) Representative immunohistochemical detection of YAP, DKK1, and CTGF in matched normal liver and HCC on a tissue microarray. (B) Pharmacologic inhibition of DKK1 reduces YAP and CTGF expression in Hep3B xenograft tumors. IHC was performed on Hep3B xenograft tumors to assess the effect of DKK1 inhibition on YAP and CTGF protein expression. Mice bearing *DKK1*-high Hep3B tumors were treated once daily for 10 consecutive days with either vehicle (*n* = 5) or the DKK1 inhibitor WAY-262611 (16 mg/kg in 200 μl; oral gavage; *n* = 5). After 10 d of oral administration, xenograft tumors were harvested and analyzed by IHC, revealing reduced YAP and CTGF staining in the WAY-262611-treated group compared with vehicle controls. Representative images are shown. (C) qPCR analysis of Hep3B cells transfected with DKK1 siRNA (*siDKK1*) or control siRNA (*siCtrl*) showing reduced expression of the YAP/TEAD target genes *CTGF*, *CYR61*, and *ANKRD1* upon DKK1 knockdown. mRNA levels were normalized to β-actin and expressed relative to the *siCtrl* group. Data are shown as mean ± SD. (D) Immunofluorescence analysis of Hep3B DKK1-KO cells showing that treatment with recombinant human DKK1 (rhDKK1; 200 ng/ml) promotes YAP nuclear localization compared with untreated cells. YAP nuclear enrichment was quantified as the nuclear-to-cytoplasmic (Nuc/Cyto) fluorescence intensity ratio (*n* = 15 cells), revealing a significant increase upon rhDKK1 treatment (*P* < 0.05). (E) Treatment with MG132 (25 μM) to block proteasomal degradation clearly showed an increase in YAP phosphorylation following DKK1 knockdown in Hep3B cells. Hep3B cells were transfected with *siDKK1* for 48 h and then treated with the proteasome inhibitor MG132 (25 μM) for 6 h prior to harvest. Whole-cell lysates were analyzed by Western blotting to assess total YAP and phosphorylated YAP (p-YAP). YAP phosphorylation was quantified as the ratio of p-YAP band intensity to total YAP band intensity (*n* = 3 independent experiments). Data are presented as mean ± SD. (F) Schematic model illustrating Hippo pathway inhibition by LRP6 *O*-GlcNAcylation. Our previous work showed that OGT binds LRP6 and catalyzes its *O*-GlcNAcylation, which enhances LRP6–Merlin association [7]. Under these conditions, Merlin is sequestered by LRP6 rather than engaging LATS1/2, thereby limiting Hippo kinase activation, reducing YAP phosphorylation, and promoting YAP stabilization and transcriptional activation. (G) Schematic representation of putative LRP6 *O*-GlcNAcylation sites. Putative *O*-GlcNAcylation sites on LRP6 (T1466, S1533, and S1575) were identified by mass spectrometry and are indicated in the schematic. (H) DKK1-CM induces LRP6 *O*-GlcNAcylation through T1466 and S1533. LRP6 *O*-GlcNAcylation was analyzed using a sWGA bead pull-down assay, which enriches *O*-GlcNAc-modified proteins by binding terminal GlcNAc residues. To test whether the predicted modification sites are required for DKK1-dependent *O*-GlcNAcylation, alanine-substitution mutants of LRP6 (T1466A, S1533A, and S1575A) were generated and cells were stimulated with DKK1-CM. sWGA-enriched fractions were subjected to immunoblotting for LRP6, showing that DKK1-CM-induced LRP6 *O*-GlcNAcylation was reduced in the T1466A and S1533A mutants compared with LRP6 WT. (I) T1466 is required for LRP6-mediated suppression of Hippo pathway activity. Hippo pathway was evaluated by Western blotting in cells overexpressing LRP6 WT or alanine-substitution mutants at the putative *O*-GlcNAcylation sites (LRP6 T1466A, LRP6 S1533A, and LRP6 S1575A). Overexpression of LRP6 WT reduced phosphorylation of LATS1 and YAP, consistent with decreased Hippo pathway activity. In contrast, the LRP6 T1466A mutant overexpression failed to reduce LATS1 and YAP phosphorylation, indicating that Thr^1466^ is necessary for LRP6-dependent regulation of Hippo signaling. (J) DKK1-induced YAP-TEAD transcriptional activity requires LRP6 Thr^1466^. Huh7 LRP6-KO cells were reconstituted with an empty control vector, LRP6 WT, or the LRP6 T1466A mutant and then treated with Ctrl-CM or DKK1-CM (*n* = 3). YAP-TEAD transcriptional activity was measured using an 8×GTIIC luciferase reporter. While DKK1-CM treatment significantly increased YAP-TEAD reporter activity in LRP6 WT-overexpressing cells, the corresponding change in LRP6 T1466A-overexpressing cells was modest and did not reach statistical significance. Data are presented as mean ± SD, and *P* values are indicated. (K) PI3K-PIP_3_ signaling contributes to DKK1-induced LRP6 *O*-GlcNAcylation. To test whether PIP_3_ generation is required for DKK1-dependent LRP6 *O*-GlcNAcylation, HEK293T cells were pretreated with the PI3K inhibitor wortmannin (100 nM) for 4 h prior to stimulation with DKK1-CM. LRP6 *O*-GlcNAcylation was then assessed by sWGA bead pull-down, which enriches *O*-GlcNAc-modified proteins. Wortmannin pretreatment attenuated DKK1-CM-induced LRP6 *O*-GlcNAcylation. In parallel, DKK1-CM-induced PIP_3_ pathway activation was monitored by immunoblot detection of AKT phosphorylation as a downstream readout of PI3K/PIP_3_ signaling. (L) Schematic illustrating the working model in which DKK1-induced PIP_3_ production promotes recruitment of OGT to the plasma membrane, thereby enhancing LRP6 *O*-GlcNAcylation. (M) rhDKK1 promotes plasma membrane localization of OGT. Immunofluorescence staining was performed to examine OGT subcellular localization following treatment with rhDKK1 (200 ng/ml) for 30 min (*n* = 6). White arrows denote regions with membrane-enriched OGT signal. The region showing OGT membrane localization (outlined by the white box) is enlarged and presented in the left panel. (N) OGT membrane localization is required for LRP6 *O*-GlcNAcylation. Left, schematic of OGT fusion constructs used to dissect the role of membrane targeting: full-length EGFP-OGT; EGFP-OGT ΔPPO, which lacks the PPO domain; and TmEGFP-OGT ΔPPO, a membrane-targeted OGT ΔPPO fusion containing the LRP6 signal peptide and transmembrane domain. Right, LRP6 *O*-GlcNAcylation was assessed by immunoprecipitation followed by immunoblotting in HEK293T cells overexpressing EGFP-OGT, EGFP-OGT ΔPPO, or TmEGFP-OGT ΔPPO. Overexpression of EGFP-OGT markedly increased LRP6 *O*-GlcNAcylation, whereas EGFP-OGT ΔPPO overexpression failed to do so. Forced membrane targeting of OGT ΔPPO (TmEGFP-OGT ΔPPO) overexpression restored LRP6 *O*-GlcNAcylation, indicating that OGT promotes LRP6 *O*-GlcNAcylation through PPO domain-dependent membrane localization. (O) EGFP–PPO overexpression attenuates DKK1-CM-induced LRP6 *O*-GlcNAcylation and reduces YAP levels. Upper, schematic of the EGFP–PPO construct used to mask PIP_3_; EGFP–PPO contains the OGT PPO domain fused to the C terminus of EGFP. Lower, control or EGFP–PPO-overexpressing Huh7 cells were treated with DKK1-CM, and LRP6 *O*-GlcNAcylation was analyzed by sWGA bead pull-down. DKK1-CM increased LRP6 *O*-GlcNAcylation in control cells, whereas this induction was attenuated by EGFP–PPO overexpression. EGFP–PPO overexpression also reduced YAP protein levels, consistent with suppression of DKK1-driven signaling. (P) EGFP–PPO overexpression increases YAP phosphorylation under proteasome inhibition in Huh7 cells. To assess the effect of EGFP–PPO overexpression on YAP phosphorylation, Huh7 cells overexpressing EGFP–PPO were treated with the proteasome inhibitor MG132 (25 μM, 6 h) to stabilize p-YAP prior to harvest. Whole-cell lysates were analyzed by Western blotting for total YAP and p-YAP. EGFP–PPO overexpression increased YAP phosphorylation, quantified as the p-YAP/total YAP band-intensity ratio from *n* = 3 independent experiments. Data are presented as mean ± SD. (Q) EGFP–PPO overexpression suppresses DKK1-induced migration and invasion of Huh7 cells. Transwell migration and Matrigel invasion assays were performed to determine whether DKK1-CM promotes Huh7 cell motility and whether this effect is altered by EGFP–PPO overexpression. Ctrl-CM or DKK1-CM was applied to Huh7 cells expressing the indicated EGFP constructs, including EGFP–PPO. DKK1-CM increased both migration and invasion compared with Ctrl-CM; however, this DKK1-CM-induced enhancement was attenuated in EGFP–PPO-overexpressing Huh7 cells. Migration and invasion were quantified as the stained area relative to the total membrane area in the Transwell assays. (R) EGFP–PPO overexpression suppresses clonogenic growth of Hep3B and Huh7 cells. Colony formation assays were performed in Hep3B and Huh7 cells to assess the effect of the EGFP–PPO construct on long-term proliferative capacity. EGFP–PPO overexpression reduced colony-forming ability compared with controls. Colonies were quantified using ImageJ by measuring (i) the number of colonies and (ii) total colony area normalized to the total well area. Data are presented as mean ± SD. (S) EGFP–PPO overexpression selectively reduces YAP protein levels in Hep3B cells without globally altering *O*-GlcNAcylation. Immunoblotting was performed in human hepatocellular carcinoma cell line Hep3B, human embryonic kidney cell line HEK293, and human normal liver cell line MIHA to evaluate the effect of EGFP–PPO overexpression on YAP protein abundance and on overall cellular *O*-GlcNAcylation. EGFP–PPO overexpression markedly reduced YAP protein levels in Hep3B cells, but had minimal effect on YAP levels in the nontumor cell lines (HEK293 and MIHA). In contrast, total protein *O*-GlcNAc levels remained largely unchanged across all 3 cell lines, indicating that EGFP–PPO does not broadly perturb global *O*-GlcNAcylation under these conditions. (T) EGFP–PPO overexpression selectively suppresses proliferation in Hep3B cells as assessed by Ki-67 staining. Cell proliferation was evaluated by Ki-67 immunofluorescence in Hep3B cells and in nontumor cell lines (HEK293 and MIHA) following EGFP–PPO overexpression. EGFP–PPO overexpression had minimal effects on Ki-67 staining in HEK293 and MIHA cells, but markedly reduced Ki-67 positivity in Hep3B cells, suggesting a tumor-selective antiproliferative effect. Cells outlined in white denote EGFP–PPO-overexpressing cells. *ANKRD1*, ankyrin repeat domain 1; CM, conditioned medium; CKAP4, cytoskeleton associated protein 4; CRDs, cysteine-rich domains; *CTGF*, connective-tissue growth factor; *CYR61*, cellular communication network factor 1; DKK1, dickkopf-1; EGFP, enhanced green fluorescent protein; GlcNAc, N-acetylglucosamine; HCC, hepatocellular carcinoma; IHC, immunohistochemical; KO, knockout; LATS1/2, large tumor suppressor kinase 1/2; LIHC, liver hepatocellular carcinoma; LRP6, low-density lipoprotein receptor-related protein 6; LRP6m5, LRP6 containing alanine substitutions at 5 PPPS/TP motifs; MST1/2, mammalian STE20-like protein kinase 1/2; Merlin, moesin–ezrin–radixin-like protein; NO. of colonies, number of colonies; OGA, *O*-GlcNAcase; *O*-GlcNAc, *O*-linked N-acetylglucosamine; OGT, *O*-GlcNAc transferase; PLA, proximity ligation assay; PIP_3_, phosphatidylinositol (3,4,5)-triphosphate; PTEN, phosphatase and tensin homolog; PI3K, phosphoinositide 3-kinase; PPO, phosphoinositide-interaction domain of OGT; p-YAP, phosphorylated YAP; qPCR, quantitative polymerase chain reaction; rhDKK1, recombinant human DKK1; ROTAC–LRP6, R-spondin and LRP6 targeting chimera; siRNA, small interfering RNA; SOST1, sclerostin1; sWGA, succinylated wheat germ agglutinin; TAZ, PDZ binding motif; TCGA, The Cancer Genome Atlas; TEAD, TEA domain transcription factor; TMA, tissue microarray; WT, wild-type; YAP, yes-associated protein.

To elucidate how DKK1 disrupts the LATS1–Merlin interaction, we tested sclerostin 1 (SOST1), another low-density lipoprotein receptor-related protein 6 (LRP6)-binding Wnt antagonist, but it failed to induce YAP nuclear localization (Fig. S6A to C). Treatment of rhDKK1 and overexpression of LRP6 wild-type (LRP6 WT) or an LRP6m5 mutant carrying alanine substitutions in the 5 PPPS/TP motifs required for canonical Wnt activation revealed that both forms suppressed Hippo signaling and activated YAP (Fig. S6D to J). Moreover, both LRP6m5 overexpression and β-catenin knockdown reduced active β-catenin levels, but decreased YAP phosphorylation was observed only with LRP6m5 overexpression (Fig. S6K and L). Notably, TCGA analysis showed a positive *DKK1*–YAP target gene correlation only in *LRP6*-high HCCs (Fig. S7A), suggesting that DKK1 modulates Hippo signaling independently of canonical Wnt signaling but in an LRP6-dependent manner. Consistently, R-spondin and LRP6 targeting chimera (ROTAC–LRP6)-mediated LRP6 degradation [6] reduced YAP levels and inhibited DKK1-CM-induced migration and invasion (Fig. S7B to E), confirming that DKK1 acts through LRP6 to promote HCC progression.

*O*-GlcNAcylation of LRP6 enhances its interaction with Merlin, disrupting the Merlin–LATS1 complex and suppressing LATS1 phosphorylation (Fig. 1F) [7]. DKK1 increased LRP6 *O*-GlcNAcylation and LRP6–*O*-GlcNAc transferase (OGT) binding in HCC cells, along with suppression of the Hippo signaling (Fig. S8). Time-course experiments showed that DKK1-induced LRP6 *O*-GlcNAcylation preceded increased LRP6–Merlin interaction and decreased LATS1–Merlin interaction (Fig. S9). Domain mapping revealed that the region surrounding the PPPS/TP motif A of LRP6 is critical for OGT binding and Hippo signaling suppression (Fig. S10). Among the DKK1-induced *O*-GlcNAcylation sites identified by mass spectrometry, T1466 was essential for LRP6-dependent Hippo regulation (Fig. 1G to J, Fig. S11, and Table S1).

DKK1 has also been reported to promote HCC progression via cytoskeleton-associated protein 4 (CKAP4)-mediated phosphatidylinositol (3,4,5)-triphosphate (PIP_3_) formation [1,8]. Using DKK1 domain-deletion constructs, we found that both the CKAP4-binding (CRD1) and LRP6-binding (CRD2) domains were required for LRP6 *O*-GlcNAcylation and YAP nuclear localization (Fig. S12A to D). Modulation of PIP_3_ formation via wortmannin treatment, phosphoinositide 3-kinase (PI3K) overexpression, or phosphatase and tensin homolog (PTEN) knockdown altered the OGT–PIP_3_ interaction and LRP6 *O*-GlcNAcylation (Fig. 1K and Fig. S12E to K). Because OGT associates with the plasma membrane through its PIP_3_-binding PPO (phosphoinositide-interaction domain of OGT) domain [9], we tested whether DKK1 enhances LRP6 *O*-GlcNAcylation by promoting OGT membrane localization. DKK1 promoted OGT membrane localization without altering total *O*-GlcNAc levels or OGT *O*-GlcNAcylation (Fig. 1L and M and Figs. S9A and S13A to D). OGT lacking the PPO domain failed to induce LRP6 *O*-GlcNAcylation, whereas a membrane-tethered OGT chimera restored this modification and enhanced Hippo suppression (Fig. 1N and Fig. S13E to J). Thus, OGT membrane localization is essential for LRP6 *O*-GlcNAcylation and Hippo signaling suppression.

We next examined whether expression of the PPO domain could serve as a competitive PIP_3_-masking strategy to inhibit DKK1- and PIP_3_-mediated LRP6 *O*-GlcNAcylation (Fig. S14A). The ectopically expressed enhanced green fluorescent protein (EGFP)–PPO construct bound to PIP_3_ (Fig. S14B) and attenuated DKK1-induced LRP6 *O*-GlcNAcylation, reduced YAP protein levels, and increased YAP phosphorylation (Fig. 1O and P). Moreover, EGFP–PPO overexpression markedly suppressed migration, invasion, and colony formation of HCC cell lines (Fig. 1Q and R and Fig. S14C).

OGT-mediated *O*-GlcNAcylation is frequently up-regulated in cancer cells, where it promotes oncogenic signaling through modification of multiple substrates [10]. However, conventional OGT inhibitors lack tumor specificity and can be toxic. Consistently, the pan-OGT inhibitor OSMI-1 reduced YAP protein and global *O*-GlcNAcylation in HEK293, Huh7, and Hep3B cells (Fig. S15A). In contrast, EGFP–PPO overexpression decreased YAP levels in Hep3B, Huh7, and PTEN-deficient U87-MG and PC-3 cells, but not in HEK293 or MIHA cells, while minimally affecting total *O*-GlcNAcylation across all lines (Fig. 1S and Fig. S15B and C). Similarly, when PPO overexpression was induced using a TetON system, YAP levels were reduced in Hep3B cells but not in HEK293 cells (Fig. S15D and E). Moreover, overexpression of EGFP–PPO reduced Ki-67 levels in Hep3B but not in HEK293 or MIHA cells (Fig. 1T), indicating selective reactivation of Hippo signaling and growth suppression in HCC cells.

In summary, we identified DKK1, which is frequently elevated in HCC, as a potent, Wnt-independent suppressor of Hippo signaling that operates through LRP6. DKK1 engages both LRP6 and CKAP4 to drive PI3K–PIP_3_-dependent recruitment of OGT to the plasma membrane, where OGT *O*-GlcNAcylates LRP6 at T1466. This modification disrupts the Merlin–LATS1 complex, reduces LATS1 and YAP phosphorylation, and promotes YAP accumulation and nuclear TEAD activity, thereby overriding contact inhibition. Overall, our study established a mechanistic bridge between metabolic signaling and growth control, showing how DKK1 could hijack the nutrient-sensing *O*-GlcNAc machinery to circumvent contact inhibition and sustain tumor proliferation (Fig. S16). Although further in vivo validation is needed, our work provides proof-of-concept therapeutic strategies to reactivate Hippo signaling and suppress malignant phenotypes.

## Ethics Approval and Consent to Participate

The study was conducted in accordance with the ethical principles of the University of Seoul. All animal experiments were conducted in accordance with the Guide for the Care and Use of Laboratory Animals and approved by the Institutional Animal Care and Use Committee (IACUC) of Ajou University Medical Center (approval no. IACUC-2022-0049, Suwon, South Korea).

## Data Availability

No datasets were generated during the current study. The data that support the findings of this study are available from the corresponding author upon reasonable request.
