# Supplementary material for: DKK1 Suppresses Hippo Signaling via PIP3–OGT–LRP6 O-GlcNAcylation in Hepatocellular Carcinoma
Source: Cancer Commun (Lond). 2026 Apr 27;46:0022. doi: 10.34133/cancomm.0022 (PMC13112786; doi:10.34133/cancomm.0022)
Supplement: Supplementary 1 — Materials and Methods Tables S1 to S5 Figs. S1 to S16 [file cancomm.0022.f1.zip › CANCOMM-D-25-00137-Supplementary-Final.docx]

**Supplementary Materials for:**

**DKK1 Suppresses Hippo Signaling via PIP_3_-OGT-LRP6 *O*-GlcNAcylation in Hepatocellular Carcinoma**

Ukjin Lee^1^, Jung Woo Eun^2^, Taehee Kim^1^, Hyewon Shim^1^, Yunho Choi^1^, Hyeryeon Jung^3^, Jaewoo Mo^4^, Soon Sun Kim^2^, Geum Ok Baek^2^, Moon Gyeong Yoon^2^, Hyeyoon Lee^5^, Wonhwa Lee^6^, Wantae Kim^1^, Junil Kim^4,7^, Eugene C. Yi^3^, Seung Up Kim^8^, Jae Youn Cheong^2, *^, Eek-Hoon Jho^1, *^

^1^Department of Life Science, University of Seoul, Seoul 02504, Republic of Korea

^2^Department of Gastroenterology, Ajou University School of Medicine, Suwon 16499, Republic of Korea

^3^Department of Molecular Medicine and Biopharmaceutical Sciences, Seoul National University, Seoul 03080, Republic of Korea

^4^Department of Bioinformatics, Soongsil University, Seoul 06978, Republic of Korea

^5^Division of Molecular Embryology, Center for Molecular Biology of Heidelberg University-German Cancer Research Center Alliance, Heidelberg 69120, Germany

^6^Department of Chemistry, Sungkyunkwan University, Suwon 16419, Republic of Korea

^7^School of Systems Biomedical Science, Soongsil University, Seoul 06978, Republic of Korea

^8^Department of Internal Medicine, Yonsei University College of Medicine, Seoul 03722, Republic of Korea

^*^Corresponding authors:

Eek-Hoon Jho, Department of Life Science, University of Seoul, Seoul 02504, Republic of Korea, E-mail: [ej70@uos.ac.kr](mailto:ej70@uos.ac.kr);

Jae Youn Cheong, Department of Gastroenterology, Ajou University School of Medicine, Suwon 16499, Republic of Korea, E-mail: jaeyoun620@gmail.com

**Supplementary Materials and Methods**

**Single-cell RNA-seq data preprocessing and analysis**

Publicly available single-cell RNA-seq data were obtained from the Gene Expression Omnibus (GEO; accession GSE149614), and we used the author-provided mapped raw unique molecular identifier (UMI) count matrices (alignment performed in the original study) as input for all analyses. Quality control filtering removed cells with < 1,000 total UMI counts or < 200 detected genes as likely empty droplets. Cells with >7,000 detected genes were flagged as putative doublets/multiplets and excluded. Cells with a mitochondrial gene fraction exceeding 20%, interpreted as stressed/dying or otherwise low-quality cells, were also removed. All subsequent preprocessing and analyses were performed using Scanpy (v1.10.4) [PMID: 29409532]. Filtered count matrices were library-size normalized and log-transformed prior to downstream analyses. Highly variable genes (HVGs) were identified using Scanpy’s highly_variable_genes with default settings and the n_top_genes option, specifying batch_key='original_sample_id' to prioritize features that are consistently variable across samples. The expression matrix was then scaled (zero mean, unit variance), followed by principal component analysis (PCA) using 50 components. To mitigate sample-specific batch effects, we constructed batch-balanced k-nearest neighbor (BBKNN) graphs (v1.6.0) [PMID: 31400197]. The BBKNN graph served as the basis for computing Uniform Manifold Approximation and Projection (UMAP) embeddings, and cells were subsequently clustered with the Leiden algorithm. Cell-type annotations were assigned using canonical marker gene expression and corroborated by differential expression (Wilcoxon rank-sum test).

**Plasmids, siRNAs, sgRNAs, and reagents**

8×GTIIC-Luc, pcDNA3-myc-LATS1, pcDNA3-Flag-hNF2 were purchased from Addgene (plasmid #34615, #41156 and #107150, respectively). pSuper‐TOP and Flag-MST1 were kindly provided by Dr. Dae‐Sik Lim (Korea advanced institute of science and technology, Korea). Meanwhile, pCS2‐VSVG-LRP6, pCS2‐LRP6‐EGFP, pRK-hSOST, pLVX-TetOne-Flag-hTOX-IRES-GFP, and pCS2-RSPO2-ΔC-FA -Flag (ROTAC-Ctrl), were generously provided by Dr. Xi He (Harvard Medical School, USA), Dr. Akira Kikuchi (Osaka University, Japan), Dr. Hee-Jung Choi (Seoul National University, Korea), Dr. Wantae Kim (University of Seoul, Korea), and Dr. Christof Niehrs (German Cancer Research Center, Germany), respectively. To generate pCS2-RSPO2-ΔC-FA-GS linker-CRD2-Flag (ROTAC-LRP6), which targets LRP5/6 via the cysteine-rich domain2 (CRD2) of DKK1, the CRD2 sequence of DKK1 was amplified using PCR-based gene slicing and inserted between the GS linker and Flag in the ROTAC-Ctrl construct. Flag-OGT was generously provided by Dr. Jin Won Cho (Yonsei University, Korea). To generate EGFP-OGT, the coding sequence of OGT was amplified from Flag-OGT and then inserted into EGFP-C1 (Original cat. #6084-1, Clontech). To generate EGFP-OGTΔPPO, the EGFP-OGT sequence lacking the PPO domain was amplified by PCR, followed by polynucleotide kinase (PNK) treatment and a ligation step for cloning. To generate TmEGFP-OGT and TmEGFP-OGTΔPPO, the N-terminal sequence of LRP6, from the start codon up to the transmembrane domain, was amplified using PCR-based gene slicing and inserted upstream of the N-terminus of EGFP-OGT and EGFP-OGTΔPPO. To generate EGFP-PPO and pLVX-TetOne-PPO-IRES-GFP, the PPO domain sequence of OGT was amplified from Flag-OGT and cloned into the EGFP-C1 and pLVX-TetOne-IRES-GFP vectors, respectively. To generate a single guide RNA (sgRNA) expressing vector, inserting sgRNA sequence was designed using the CHOPCHOP (<https://chopchop.cbu.uib.no>) and each sgRNA was inserted into the lentiCRISPR2 vector (Cat. #52961, Addgene). siRNAs were purchased from Genolution (Korea) and Genepharma (China). Materials and sequences of the siRNAs and sgRNAs are provided in Supplementary Tables S2 and S3, respectively.

**Cell lines used in this study**

The human embryonic kidney cell lines HEK293 and HEK293T, the human normal liver cell line MIHA, the human hepatocellular carcinoma cell lines Huh7, HepG2, and Hep3B, the human glioblastoma cell line U87-MG, and the human prostate cancer cell line PC-3 were used in this study.

**Cell culture and transfection**

The human hepatocellular carcinoma cell lines Huh7, HepG2 and the human prostate cancer cell line PC-3 were cultured in Roswell Park Memorial Institute (RPMI) 1640 medium (Cat. #SH30027, Hyclone) supplemented with 10% fetal bovine serum (FBS; Cat. #26140-079, Gibco) and 1×antibiotic-antimyotic (Cat. #15240-062, Gibco) at 37°C in a humidified incubator with 5% CO₂. The human embryonic kidney cell lines HEK293, HEK293T, the human normal liver cell line MIHA, the human hepatocellular carcinoma cell lines Hep3B and the human glioblastoma cell lines U87-MG were cultured in Dulbecco's Modified Eagle Medium (DMEM; Cat. #10-013-CV, Corning) supplemented with 10% FBS and 1× antibiotic-antimyotic at 37°C in a humidified incubator with 5% CO₂. For transfection of HEK293T cells, the calcium phosphate method was used. All other cell lines were transfected using lipofectamine 2000 (Cat. #11668019, Thermo Fisher Scientific) or lipofectamine 3000 (Cat. #L3000015, Thermo fisher scientific) according to the manufacturer's instructions. siRNA transfection was performed using lipofectamine RNAiMAX (Cat. #13778150, Thermo Fisher Scientific).

**Lentiviral transduction and generation of genome-edited cells**

HEK293T cells were transfected with a plasmid mixture containing a lentiviral vector encoding the sgRNA targeting the gene of interest, the packaging vector psPAX2, and the envelope vector pVSVG. The following day, the viral supernatant was collected as viral-conditioned media from the HEK293T cells. Viral particles were precipitated using PEG-it according to the manufacturer’s instructions and subsequently resuspended in cold PBS (4°C). The virus-containing PBS was added to the cultured target cell lines along with Polybrene. After 1 day, infected cells were subjected to puromycin selection. Three days later, surviving cells were single-cell sorted into 96-well plates using FACS.

**Immunoblotting and immunoprecipitation**

Cells were harvested by scraping with a cell scraper and centrifuged at 12,500 × g for 5 minutes in phosphate-buffered saline (PBS) at 4 °C. The resulting pellets were resuspended in lysis buffer containing 20 mmol/L Tris (pH 7.4), 150 mmol/L NaCl, 1% Triton X-100, 1 mmol/L EDTA, 1 mmol/L EGTA, 2.5 mmol/L sodium pyrophosphate, 1 mmol/L β-glycerophosphate, 1 mmol/L sodium orthovanadate, 1 mmol/L phenylmethylsulfonyl fluoride (PMSF), and 1 μg/mL leupeptin. For experiments involving the detection of protein *O*-GlcNAcylation, 10 μmol/L PUGNAc (an *O*-GlcNAcase inhibitor) was additionally included in the lysis buffer. After incubation on ice for 30 minutes, the lysates were centrifuged at 13,000 × g for 10 minutes at 4 °C. The supernatants were collected and used for immunoblotting and immunoprecipitation analyses. For immunoblotting, protein concentrations of each lysate were determined using the Bradford assay. Equal amounts of protein were mixed with 4× SDS loading buffer (200 mmol/L Tris, pH 6.8; 8% SDS; 0.05% bromophenol blue; 40% glycerol; and 200 mmol/L β-mercaptoethanol) and boiled for 10 minutes. The samples were separated by SDS-PAGE and transferred onto PVDF membranes. Membranes were incubated with primary antibodies diluted in 5% skim milk or bovine serum albumin (BSA) overnight at 4°C. The following day, membranes were washed five times in 1× TBST buffer for 10 minutes per wash. After washing, membranes were incubated with secondary antibodies diluted in 5% skim milk for 1 hour at room temperature, followed by three washes in 1× TBST buffer for 15 minutes each. ECL (ELPIS or Millipore) was applied to the membranes, and signals detection was performed using X-ray film or MicroChemi 4.2 (DNR Bio-ImagingSystems). For immunoprecipitation, cell lysates were incubated with the primary antibodies overnight at 4°C with rotator. Protein A/G plus agarose beads (Fast Flow, 50% slurry, Millipore) were then added and incubated for 1 hour at 4°C with rotator. The beads were washed five times with lysis buffer for 10 minutes each at 4°C. Finally, the beads were boiled in 4× SDS loading buffer for 10 minutes before analysis by SDS-PAGE. The antibodies used in immunoblotting and immunoprecipitation experiments are listed in Supplementary Table S4.

**Immunofluorescence analysis**

HEK293, Huh7, or Hep3B cells were seeded onto glass coverslips in 6-well plates and transfected with plasmids or siRNAs as indicated in each panel. For immunofluorescence analysis, the coverslips were transferred to 24-well plates, fixed with 4% paraformaldehyde (Cat. #P2031, Biosesang) for 20 minutes at room temperature, and briefly washed three times with PBS. After washing, cells were permeabilized with 0.1% Triton X-100 in PBS for 15 minutes at room temperature and then washed three times with PBS. Blocking was performed using 5% BSA in PBS for 1 hour at room temperature, followed by incubation with primary antibodies diluted in 5% BSA in PBS overnight at 4 °C with gentle rocking. After primary antibody incubation, cells were washed three times with 1% BSA in PBS for 10 minutes each. Cells were then incubated with secondary antibodies diluted in 5% BSA in PBS for 1 hour at room temperature with gentle rocking. Following incubation, cells were washed three times with 1% BSA in PBS for 10 minutes each. DAPI staining (300 ng/mL; Cat. #D9542, Sigma Aldrich) was performed during the second wash step. Finally, glass coverslips were mounted onto microscope slides using mounting solution (Cat. #0100-01, Southern Biotech), and fluorescence images were acquired using an LSM 800 confocal microscope (Zeiss) at the Center for Research Facilities, University of Seoul. The primary and secondary antibodies used in immunofluorescence experiments are listed in Supplementary Table S4.

**Subcellular fractionation analysis**

Subcellular fractionation was performed as previously described. Cells seeded in culture dishes were harvested using a cell scraper and centrifuged at 14,000 × g for 5 min at 4°C to collect the cell pellet. A portion of the pellet was used for preparing total cell lysates. The remaining cells were resuspended in S100/P100 buffer supplemented with 1 mmol/L phenylmethylsulfonyl fluoride and 1 μg/mL leupeptin, followed by incubation for 20 min on ice. The cell suspension was then passed through a 26-gauge needle 20 times. The homogenate was centrifuged at 1,000 × g for 2 min at 4°C to remove nuclei (pellet), and the resulting supernatant was further centrifuged at 20,000 × g for 60 min at 4°C. The pellet (membrane fraction) and supernatant (cytosolic fraction) were collected separately. Membrane, cytosolic, and total lysates were subjected to immunoblotting analysis as described above.

**RNA extraction, cDNA synthesis and quantitative real-time PCR**

Total RNA was extracted using TRI Reagent (Cat. #BLS-TR118, Molecular research center) according to the manufacturer’s protocol. Briefly, culture medium was removed, and cells were treated with 1 mL of TRI Reagent for 5 min on a rocker at 25°C. Lysates were transferred to tubes, and 0.2 mL of chloroform was added, followed by vigorous mixing and incubation for 3 min at 25°C. Samples were centrifuged at 12,000 × g for 15 min at 4°C, and the upper aqueous phase (0.2–0.3 mL) was collected. RNA was precipitated by adding 0.5 mL of isopropyl alcohol and incubating for 10 min at 25°C, followed by centrifugation at 12,000 × g for 10 min at 4°C. Pellets were washed with 75% ethanol and centrifuged at 7,500 × g for 5 min at 4°C. RNA pellets were air-dried and dissolved in DEPC-treated water by pipetting and incubated at 60°C for 10 min. Complementary DNA (cDNA) was synthesized using the ReverTra Ace® qPCR RT Kit (Cat. #FSQ-201, Toyobo) according to the manufacturer's instructions. Quantitative real-time PCR was performed using THUNDERBIRD® SYBR® qPCR Mix (Cat. #QPS-201, Toyobo). The threshold cycle (Ct) value of β‐ACTIN or Gapdh was used to normalize gene expression levels, and relative mRNA expression was calculated using the ΔΔCt method. Primers used in qPCR analysis are listed in Supplementary Table S5

**Dual-luciferase reporter assay**

HEK293T, HEK293, Huh7, and Hep3B cells were seeded in 12-well plates and transfected with a mixture containing the desired reporter plasmid (500 ng of either 8×GTIIC-Luc or pSuperTOP-Luc per well), thymidine kinase promoter-driven Renilla luciferase plasmid (pRL-TK, 50 ng per well), and the requisite plasmid constructs. After 24 hours, cells were lysed in 1× passive lysis buffer (PLB; Cat. #E194A, Promega) for 15 minutes. Lysates were centrifuged at 14,000 × g for 10 minutes at 4 °C, and 10 μL of the resulting supernatant was used for the luciferase assay. Firefly and Renilla luciferase activities were measured using the Dual-Luciferase® Reporter Assay System (Cat. #E1960, Promega) according to the manufacturer’s instructions. Luminescence was detected using a GloMax® 20/20 luminometer (Promega). Firefly luciferase activity was normalized to Renilla luciferase activity.

**Proximity-ligation assay**

Cells grown on coverslips were fixed with 4% paraformaldehyde for 20 minutes and washed three times with PBS. Permeabilization was performed with 0.1% Triton X-100 in PBS for 15 minutes, followed by three additional PBS washes. Cells were then incubated with the blocking solution provided in the NaveniFlex™ Cell MR kit (Cat. #60025, Navinci) for 1 hour at 37°C. Subsequent steps were performed according to the manufacturer's protocol. For nuclear staining, cells were incubated with DAPI staining solution prepared in 1% BSA for 10 minutes. After staining, the mounting solution (Cat. #0100-01, Southern Biotech) was applied to a glass slide, and the coverslip was placed on top. Samples were imaged the following day using an LSM 800 confocal microscope (Zeiss). Quantification of immunofluorescence signals was performed using ImageJ and GraphPad Prism software. The primary antibodies used are listed in Supplementary Table S4.

**Succinylated Wheat Germ Agglutinin (sWGA) pull-down assay**

For the sWGA pull-down assay, cells under each condition were lysed on ice for 30 min in lysis buffer containing 20 mmol/L Tris (pH 7.4), 150 mmol/L NaCl, 1% Triton X‐100, 1 mmol/L EDTA, 1 mmol/L EGTA, 2.5 mmol/L sodium pyrophosphate, 1 mmol/L β‐glycerophosphate, 1 mmol/L sodium orthovanadate, 1 mmol/L phenylmethylsulfonyl fluoride, and 1 μg/mL leupeptin. Lysates were cleared by centrifugation at 14,000 × g for 10 min at 4°C, and protein concentrations were determined using the Bradford assay. Equal amounts of protein were incubated with sWGA-agarose beads (Cat. #AL-1023S, Vector Laboratories) for 3 h at 4°C with rotation. Beads were then washed five times using lysis buffer, with each wash involving 15 min rotation followed by centrifugation at 3,000 × g for 2 min. After the final wash, beads were resuspended in 4× SDS loading dye (200 mmol/L Tris, pH 6.8, 8% SDS, 0.05% bromophenol blue, 40% glycerol, 200 mmol/L β‐mercaptoethanol) and boiled for 10 min before analysis. For negative control, cell lysates were preincubated with N-acetylglucosamine (Free GlcNAc; Cat. #A3286, Sigma Aldrich) prior to sWGA bead incubation.

**Identification of *O*-GlcNAc Sites of LRP6 Protein**

Tryptic-digested peptides of LRP6 were dissolved in Solvent A (0.1% formic acid in H₂O) and separated on a PepMap™ RSLC C18 column (Cat. #164942, Thermo Fisher Scientific,) using a linear gradient of 2%–38% Solvent B (0.1% formic acid in acetonitrile) over 75 min at 300 nL/min. Peptides were analyzed on an Orbitrap Fusion Lumos Tribrid mass spectrometer (Thermo Fisher Scientific) interfaced with an Easy nanoLC 1200 system. The spray voltage was set to 1.9 kV, and the heated capillary was maintained at 275°C. The instrument was operated in data-dependent acquisition (DDA) mode, with one survey MS scan followed by 10 MS/MS scans and a dynamic exclusion of 30 s. Full MS scans were acquired from 350–1600 m/z at a resolution of 120,000 with an automatic gain control (AGC) target of 4e5. MS/MS scans were acquired at a resolution of 30,000 with an AGC target of 5e4 using higher-energy collisional dissociation (HCD). When oxonium ions (m/z 204.0867, 138.0545) were detected in HCD spectra, EThcD fragmentation was triggered with charge-dependent reaction times (45.20 ms for 3 +, 25.42 ms for 4 +) and supplemental HCD activation (15%–17%). EThcD MS/MS spectra were acquired at a resolution of 30,000 with an AGC target of 1e5 and a maximum injection time of 120 ms. Spectra were searched against the UniProt human database (https://www.uniprot.org/; Release 2019_11, 186,532 entries) with a decoy strategy using Sequest HT in Proteome Discoverer 2.2. Precursor and fragment ion tolerances were set at 10 ppm and 0.02 Da, respectively. Trypsin was specified as the digestion enzyme, allowing up to two missed cleavages. Modifications included carbamidomethylation (Cys, static), HexNAc (Ser/Thr, dynamic, +203.079 Da), phosphorylation (Ser/Thr, +79.966 Da), and oxidation (Met, dynamic). False discovery rates (FDRs) were calculated using the Fixed Value PSM Validator, and results were filtered to a 1% FDR at the peptide and protein levels.

**Colony-forming assay**

For Huh7 and Hep3B cell lines, transfected cells were trypsinized and counted using the Countess™ II Automated Cell Counter (Thermo Fisher Scientific). A total of 5,000 cells were seeded into each well of a 12-well plate. Cells were incubated in DMEM or RPMI medium supplemented with 10% FBS for 5–7 days at 37°C in a humidified incubator with 5% CO₂. For cell fixation, cells were treated with 4% paraformaldehyde for 20 minutes, followed by a single wash with PBS. For colony staining, a staining solution (1% crystal violet in PBS) was added to each well and incubated for 10 minutes. The wells were then gently rinsed with tap water. Images of triplicate wells were captured using a digital camera, and both colony area and colony number were quantified using ImageJ software (v1.54g).

**Transwell migration and invasion assay**

After trypsinization, transfected cells were counted using the Countess™ II Automated Cell Counter. Huh7 cells (1 × 10⁴) and Hep3B cells (1 × 10⁵) were resuspended in 200 μL of serum-free medium or serum-free conditioned medium (CM) and seeded into the upper chamber of a polycarbonate (PC) membrane hanging insert with 8 μm pores (SPLInsert™ Hanging, SPL). The lower chamber was filled with 700 μL of the corresponding medium supplemented with 10% FBS. Cells were incubated for 16–24 hours at 37°C in a humidified incubator with 5% CO₂. After incubation, chambers were gently washed with PBS and fixed in 4% paraformaldehyde for 10 minutes. Cells were stained with 0.5% crystal violet for 5 minutes, and excess dye was removed by washing with PBS. Non-invaded cells remaining on the upper surface of the membrane were gently removed using a cotton swab. Images of three random fields per upper chamber were captured using a Nikon Eclipse Ti microscope at ×200 magnification. The area of invaded or migrated cells on the lower surface was quantified using ImageJ software. For the invasion assay, the upper chamber was pre-coated with diluted Matrigel (BD Biosciences) in serum-free medium, and the assay was performed using the same Transwell protocol.

**Subcutaneous xenograft mouse model**

All animal experiments were conducted in accordance with the Guide for the Care and Use of Laboratory Animals and approved by the Institutional Animal Care and Use Committee (IACUC) of Ajou University Medical Center (approval no. IACUC-2022-0049, Suwon, South Korea). Five-week-old female BALB/c nude mice were obtained from ORIENT BIO Inc. (Seongnam, Korea). Mice were maintained in individually ventilated cages under specific pathogen-free conditions and allowed to acclimate for one week before use.

Hep3B cells (3 × 10⁶ cells/0.1mL in Matrigel/serum-free DMEM) were injected subcutaneously into the flanks of the mice. When the tumors reached a volume of approximately 100 mm³, mice were orally administered the DKK1 inhibitor WAY-262611 (16 mg/kg/day; MedChemExpress, China). Tumor size and body weight were measured every two days using a digital caliper and a digital scale, respectively. Tumor volume was calculated according to the formula: Tumor volume (mm^3^) = 0.52 × length × width^2^

After 10 days of oral administration, mice were euthanized by CO₂ inhalation, and tumors were harvested for weighing and histological analysis. For immunohistochemistry (IHC), tumor tissues were fixed in 10% neutral-buffered formalin, embedded in paraffin, sectioned at 5 µm, and deparaffinized. Sections were incubated with human-specific primary antibodies against YAP XP (Cat. #14074S, Cell Signaling Technology; 1:200) and CTGF (Cat. #ab6992, Abcam; 1:100).

**Tissue microarray samples and Immunohistochemistry (IHC)**

Four µm-thick Tissue microarray (TMA) sections containing 67 hepatocellular carcinomas (HCCs) and 10 normal livers were purchased (TissueArray.com), deparaffinized and rehydrated. Antigens were retrieved with Tris buffer, and the sections were incubated with primary antibodies YAP XP (Cat. #14074S, Cell Signaling Technology; 1:200), DKK1 (Cat. #ab61034, Abcam; 1:100), and CTGF (Cat. #ab6992, Abcam; 1:100), for 1 hour at 37°C, and further incubated with HRP-labeled secondary antibodies. The bound antibodies were visualized using a 3,3’ diaminobenzidine chromogen and counterstained with hematoxylin. IHC scoring was determined based on the percentage of stained cells and intensity of stains by pathologists. The immunoreactivity of DKK1, YAP and CTGF was scored into four different staining intensity levels: negative, weak (1 +), moderate (2 +) and strong (3 +) positivity. Cytoplasmic staining of ≥ 2 + intensity in more than 30% of HCC cells was considered indicative of protein overexpression. Correlations between DKK1 and YAP, DKK1 and CTGF, and YAP and CTGF were assessed using the chi-squared test. A *P*-value < 0.05 was considered statistically significant.

**Statistical analysis and figure preparation.**

Statistical analyses were performed using unpaired *t*-tests and ordinary one-way analysis of variance (ANOVA) with Microsoft Excel or GraphPad Prism (v10.4.2). *P-*value less than 0.05 was considered statistically significant. Quantitative data from biological replicates are presented as mean ± standard deviation (SD). Time course tumor volume data derived from xenograft mouse models are presented as mean ± standard error of the mean (SEM), and graphical data visualization was performed using the GraphPad Prism program. And all figures were processed using Microsoft PowerPoint and Adobe Photoshop. Illustration figures were produced using Biorender (<https://www.biorender.com/>) and Microsoft PowerPoint.

**Supplementary Tables**

**Supplementary Table S1. Mass spectrometry analysis of LRP6**

| **Confidence** | **Annotated Sequence** | **Modifications** | **XCorr** |
| --- | --- | --- | --- |
| **High** | **HFAPPTTPcsTDVcDSDYAPSRR** | **C9(Carbamidomethyl); S10(HexNAc); C14(Carbamidomethyl)** | **8.33** |
| High | HFAPPTTPcStDVcDSDYAPSRR | C9(Carbamidomethyl); T11(HexNAc); C14(Carbamidomethyl) | 8.03 |
| High | HFAPPTTPcsTDVcDSDYAPSRR | C9(Carbamidomethyl); S10(HexNAc); C14(Carbamidomethyl) | 7.36 |
| High | HFAPPTTPcStDVcDSDYAPSRR | C9(Carbamidomethyl); T11(HexNAc); C14(Carbamidomethyl) | 7.00 |
| High | HFAPPTTPcsTDVcDSDYAPSRR | C9(Carbamidomethyl); S10(HexNAc); C14(Carbamidomethyl) | 6.99 |
| High | HFAPPTTPcsTDVcDSDYAPSRR | C9(Carbamidomethyl); S10(HexNAc); C14(Carbamidomethyl) | 6.95 |
| High | HFAPPTTPcsTDVcDSDYAPSRR | C9(Carbamidomethyl); S10(HexNAc); C14(Carbamidomethyl) | 6.89 |
| High | HFAPPTTPcStDVcDSDYAPSRR | C9(Carbamidomethyl); T11(HexNAc); C14(Carbamidomethyl) | 6.68 |
| High | HFAPPTTPcStDVcDSDYAPSRR | C9(Carbamidomethyl); T11(HexNAc); C14(Carbamidomethyl) | 6.64 |
| High | HFAPPTTPcStDVcDSDYAPSRR | C9(Carbamidomethyl); T11(HexNAc); C14(Carbamidomethyl) | 6.63 |
| High | HFAPPTTPcsTDVcDSDYAPSR | C9(Carbamidomethyl); S10(HexNAc); C14(Carbamidomethyl) | 6.26 |
| High | HFAPPTTPcStDVcDSDYAPSR | C9(Carbamidomethyl); T11(HexNAc); C14(Carbamidomethyl) | 6.07 |
| **High** | **sQYLSAEENYEScPPSPYTER** | **S1(HexNAc); C13(Carbamidomethyl)** | **5.92** |
| High | HFAPPTTPcsTDVcDSDYAPSRR | C9(Carbamidomethyl); S10(HexNAc); C14(Carbamidomethyl) | 5.87 |
| High | HFAPPTTPcsTDVcDSDYAPSR | C9(Carbamidomethyl); S10(HexNAc); C14(Carbamidomethyl) | 5.77 |
| High | HFAPPTTPcStDVcDSDYAPSRR | C9(Carbamidomethyl); T11(HexNAc); C14(Carbamidomethyl) | 5.61 |
| High | HFAPPTTPcStDVcDSDYAPSR | C9(Carbamidomethyl); T11(HexNAc); C14(Carbamidomethyl) | 5.61 |
| High | sQYLSAEENYEScPPSPYTER | S1(HexNAc); C13(Carbamidomethyl) | 5.38 |
| High | sQYLSAEENYEScPPSPYTER | S1(HexNAc); C13(Carbamidomethyl) | 4.55 |
| High | HFAPPTTPcsTDVcDSDYAPSR | C9(Carbamidomethyl); S10(HexNAc); C14(Carbamidomethyl) | 4.53 |
| High | HFAPPTTPcStDVcDSDYAPSR | C9(Carbamidomethyl); T11(HexNAc); C14(Carbamidomethyl) | 4.44 |
| High | HFAPPTtPcSTDVcDSDYAPSR | T7(HexNAc); C9(Carbamidomethyl); C14(Carbamidomethyl) | 4.37 |
| High | HFAPPTTPcsTDVcDSDYAPSR | C9(Carbamidomethyl); S10(HexNAc); C14(Carbamidomethyl) | 4.33 |
| High | HFAPPTTPcStDVcDSDYAPSR | C9(Carbamidomethyl); T11(HexNAc); C14(Carbamidomethyl) | 4.25 |
| High | HFAPPTTPcsTDVcDSDYAPSR | C9(Carbamidomethyl); S10(HexNAc); C14(Carbamidomethyl) | 3.98 |
| High | HFAPPTTPcStDVcDSDYAPSR | C9(Carbamidomethyl); T11(HexNAc); C14(Carbamidomethyl) | 3.80 |
| High | sQYLSAEENYEScPPSPYTER | S1(HexNAc); C13(Carbamidomethyl) | 3.61 |
| High | sQYLSAEENYEScPPSPYTER | S1(HexNAc); C13(Carbamidomethyl) | 3.51 |
| High | HFAPPTTPcsTDVcDSDYAPSR | C9(Carbamidomethyl); S10(HexNAc); C14(Carbamidomethyl) | 3.19 |
| High | HFAPPTTPcStDVcDSDYAPSR | C9(Carbamidomethyl); T11(HexNAc); C14(Carbamidomethyl) | 3.09 |
| High | sQYLSAEENYEScPPSPYTER | S1(HexNAc); C13(Carbamidomethyl) | 2.88 |
| **High** | **AHVtGASSSSSSSTK** | **T4(HexNAc)** | **2.83** |
| High | AHVtGASSSSSSSTK | T4(HexNAc) | 2.62 |

Abbreviations: LRP6, low-density lipoprotein receptor-related protein 6.

**Supplementary Table S2. List of materials**

| **Materials** | **Manufacturer** | **Catalog number** |
| --- | --- | --- |
| MGH-CP1 | Selleck Chemicals | S9735 |
| WAY-262611 | MedChemExpress | HY-11035 |
| MG132 | Sigma Aldrich | C2211 |
| Recombinant human DKK1 | Abcam | ab155623 |
| DAPI | Sigma Aldrich | D9542 |
| Wortmannin | Sigma Aldrich | W1628 |
| sWGA | Vector Laboratories | AL-1023 |
| Free-GlcNAc (N-Acetyl-D-glucosamine) | Sigma Aldrich | A8625 |
| OSMI-1 | Sigma Aldrich | SML1621 |
| Doxycycline | Sigma Aldrich | D9891 |
| SPLInsert^TM^Hanging (Transwell assay) | SPL life science | 35224 |
| 25 Culture-inserts 2 Well for self-insertion | Ibidi | 80209 |
| PLA kit (NaveniFlex Cell MR) | Navinci | 60025 |

**Supplementary Table S3. List of siRNA and sgRNA**

| siRNA and sgRNA | Sequences |
| --- | --- |
| *siGFP* | sense: 5’- GUUCAGCGUGUCCGGCGAG -3’ |
|  | antisense: 5’- CUCGCCGGACACGCUGAAC -3’ |
| *siDKK1 #1* | sense: 5’- ACUCGGUUCUCAAUUCCAACGCUAUUU -3’ |
|  | antisense: 5’- AUAGCGUUGGAAUUGAGAACCGAGUUU -3’ |
| *siDKK1 #2* | sense: 5’- GCUCUCAUGGACUAGAAAUUU -3’ |
|  | antisense: 5’- AUUUCUAGUCCAUGAGAGCUU -3’ |
| *siYAP* | sense: 5’- GACAUCUUCUGGUCAGAGA -3’ |
|  | antisense: 5’- UCUCUGACCAQGAAGAUGUC -3’ |
| *siTAZ* | sense: 5’- ACGUUGACUUAGGAACUUU -3’ |
|  | antisense: 5’- AAAGUUCCUAAGUCAACGU -3’ |
| *siLRP6* | sense: 5’- ACAUUGUUCUGCAGUUAGAUU -3’ |
|  | antisense: 5’- UCUAACUGCAGAACAAUGUUU -3’ |
| *siPTEN* | sense: 5’-GUUAGCAGAAACAAAAGGAGAUAUCAAUU -3’ |
|  | antisense: 5’-UUGAUAUCUCCUUUUGUUUCUGCUAACUU -3’ |
| *siCTNNB1* | sense: 5’-CCA AGA AGC AGA GAU GGC CCA GAA UUU -3’ |
|  | antisense: 5’-AUU CUG GGC CAU CUC UGC UUC UUG GUU -3’ |
| *Non-targeting sgRNA* | sense: 5’- UCAUGCUUGCUUGGGCAAAA -3’ |
|  | antisense: 5’- UUUUGCCCAAGCAAGCAUGA -3’ |
| *sgDKK1 #1* | sense: 5’- UACCAUCGCGACAAAGACCC -3’ |
|  | antisense: 5’- GGGUCUUUGUCGCGAUGGUA -3’ |
| *sgDKK1 #2* | sense: 5’- UACCCGGGTCUUUGUCGCGA -3’ |
|  | antisense: 5’- UCGCGACAAAGACCCGGGUA -3’ |
| *sgLRP6 #1* | sense: 5’- CUCGCGUUGGACCCUGCCGA -3’ |
|  | antisense: 5’- UCGGCAGGGUCCAACGCGAG -3’ |
| *sgLRP6 #2* | sense: 5’- UCGCGUUGGACCCUGCCGAA -3’ |
|  | antisense: 5’- UUCGGCAGGGUCCAACGCGA -3’ |
| *sgLRP6 #3* | sense: 5’- GUCCACGUAUGAAGGGAGAU-3’ |
|  | antisense: 5’- AUCUCCCUUCAUACGUGGAC-3’ |

Abbreviations: *DKK1*, dickkopf-1; *GFP*, green fluorescent protein; *LRP6*, low-density lipoprotein receptor-related protein 6; sgRNA, single guide RNA; *TAZ*, transcriptional coactivator with PDZ-binding motif; *YAP*, yes-associated protein.

**Supplementary Table S4. List of antibodies**

| **Antibodies** | **Manufacturer** | **Catalog number** |
| --- | --- | --- |
| *Primary antibodies for immunoblotting* | | |
| anti-p-YAP (S127) | Cell Signaling Technology | 4911S |
| anti-YAP | Santa Cruz | sc-101199 |
| anti-p-LATS1 (T1079) | Cell Signaling Technology | 8654S |
| anti-LATS1 | Cell Signaling Technology | 3477S |
| anti-Merlin (NF2) | Cell Signaling Technology | 12888S |
| anti-DKK1 | Cell Signaling Technology | 48367S |
| anti-LRP6 | Cell Signaling Technology | 3395S |
| anti-p-LRP6 | Cell Signaling Technology | 2568S |
| anti-*O*-GlcNAc | Invitrogen | MA1-072 |
| anti-OGT | Sigma Aldrich | O6264 |
| anti-AKT | Cell Signaling Technology | 9272S |
| anti-p-AKT (S473) | Cell Signaling Technology | 9271S |
| anti-PTEN | Cell Signaling Technology | 9559S |
| anti-Flag | Sigma Aldrich | F1804 |
| anti-GFP | Santa Cruz | sc-9996 |
| anti-VSVG | Sigma Aldrich | V4888 |
| anti-β-actin | Sigma Aldrich | A5441 |
| anti-β-tubulin | Genetex | GTX101279 |
| anti-Na ^+^ /K ^+^ - ATPase | Santa Cruz | sc-48345 |
| *Secondary antibodies for immunoblotting* | | |
| Goat anti-mouse IgG (HRP conjugate) | Jackson ImmunoResearch | 115-035-003 |
| Goat anti-rabbit IgG (HRP conjugate) | Jackson ImmunoResearch | 111-035-003 |
| Goat anti-mouse IgG (HRP conjugate, light chain specific) | Jackson ImmunoResearch | 115-035-174 |
| Mouse anti-rabbit IgG (HRP conjugate, light chain specific) | Jackson ImmunoResearch | 211-032-171 |
| *For immunoprecipitation* | | |
| anti-EGFP | Invitrogen | A-11122 |
| anti-VSVG | Sigma Aldrich | V4888 |
| anti-Flag | Sigma Aldrich | F1804 |
| anti-Merlin | Santa Cruz | sc-55575 |
| *For immunofluorescence (primary and secondary)* | | |
| anti-YAP | Cell Signaling Technology | 14074S |
| anti-OGT | Sigma Aldrich | O6264 |
| anti-Ki-67 | Abcam | Ab15580 |
| anti-Cleaved caspase3 | Cell Signaling Technology | 9661S |
| Alexa Fluor 488 goat anti-mouse | Invitrogen | A11029 |
| Alexa Fluor 488 goat anti-rabbit | Invitrogen | A11034 |
| Alexa Fluor 546 goat anti-mouse | Invitrogen | A11030 |
| Alexa Fluor 546 goat anti-rabbit | Invitrogen | A11035 |
| *For proximity-ligation assay* | | |
| anti-TEAD1 | Cell Signaling Technology | 12292S |
| anti-YAP | Santa Cruz | sc-101199 |
| anti-Merlin (NF2) | Santa Cruz | sc-55575 |
| anti-LATS1 | Cell Signaling Technology | 3477S |
| anti-LRP6 | Invitrogen | PA5-101047 |
| anti-OGT | Santa Cruz | sc-74546 |
| anti-PIP_3_ | Invitrogen | A-21328 |
| anti-EGFP | Invitrogen | A-11122 |

Abbreviations: AKT, protein kinase B; DKK1, dickkopf-1; GFP, green fluorescent protein; LATS1, large tumor suppressor kinase 1; LRP6, low-density lipoprotein receptor-related protein 6; Merlin, moesin-ezrin-radixin-like protein; NF2, neurofibromatosis type 2; OGT, *O*-GlcNAc transferase; PIP_3_, phosphatidylinositol (3,4,5)-trisphosphate; PTEN, phosphatase and tensin homolog; YAP, yes-associated protein; VSVG, vesicular stomatitis virus G glycoprotein.

**Supplementary Table S5. List of primers**

| **Primers for qPCR (Human)** | **Sequence** |
| --- | --- |
| *CTGF*-F | CTTGCGAAGCTGACCTGGAAGA |
| *CTGF*-R | CCGTCGGTACATACTCCACAGA |
| *CYR61*-F | GGAAAAGGCAGCTCACTGAAGC |
| *CYR61*-R | GGAGATACCAGTTCCACAGGTC |
| *ANKRD1*-F | GCTTTGGTTCCATTCTGCCAGTG |
| *ANKRD1*-R | CGACTCCTGATTATGTATGGCGC |
| *DKK1*-F | AAGCGCCGAAAACGCTGCAT |
| *DKK1*-R | GATCTTGGACCAGAAGTGTC |
| *YAP*-F | TAGCCCTGCGTAGCCAGTTA |
| *YAP*-R | TCATGCTTAGTCCACTGTCTGT |
| *TAZ*-F | GGCTGGGAGATGACCTTCAC |
| *TAZ*-R | CTGAGTGGGGTGGTTCTGCT |
| *LRP6*-F | CCCATGCACCTGGTTCTACT |
| *LRP6*-R | CCAAGCCACAGGGATACAGT |
| *AXIN2-F* | AGTGTGAGGTCCACGGAAAC |
| *AXIN2-R* | CTTCACACTGCGATGCATTT |
| *GAPDH*-F | TGCACCACCAACTGCTTACC |
| *GAPDH*-R | TCTTCTGGGTGGCAGTGATG |

Abbreviations: *ANKRD1*, ankyrin repeat domain 1; *AXIN2*, axis inhibition protein 2; *CTGF*, connective tissue growth factor; *CYR61*, cysteine-rich angiogenic inducer 61; *DKK1*, dickkopf-1; *GAPDH*, glyceraldehyde 3-phosphate dehydrogenase; *LRP6*, low-density lipoprotein receptor-related protein 6; *TAZ*, transcriptional coactivator with PDZ-binding motif; *YAP*, yes-associated protein.

**Supplementary Figures**

**Supplementary Figure S1. Hepatocytes in hepatocellular carcinoma tissues exhibited elevated expression of *DKK1* and YAP target genes.**

Bulk RNA-seq data from TCGA-LIHC (*n* = 358) and publicly available HCC scRNA-seq datasets (GEO; accession GSE149614) were analyzed to evaluate the association between *DKK1* expression and Hippo-YAP/TAZ-TEAD pathway activity. **(A)** Left panel: Pie chart showing the distribution of major oncogenic mutations in TCGA-LIHC. *TP53* mut LIHC were detected in 31.0% of LIHC cases, *CTNNB1* mut LIHC in 20.4%, *AXIN1* mut LIHC in 5.3%, and *TP53/CTNNB1/AXIN1* WT LIHC accounted for 43.3%. Right panel: Normalized *DKK1* mRNA expression in TCGA-LIHC tumors stratified by representative HCC driver-mutation subgroups, compared with normal liver tissue; *DKK1* expression was elevated across all mutation-defined tumor subgroups relative to normal liver. **(B)** In TCGA-LIHC, tumors were stratified into *DKK1*-high and *DKK1*-negative groups based on *DKK1* mRNA expression; the top 10% were designated as “*DKK1*-high” TCGA-LIHC tumors, and tumors with undetectable *DKK1* mRNA expression were designated as “no *DKK1”* TCGA-LIHC tumors. Differential expression analysis comparing the “*DKK1*-high” TCGA-LIHC tumors with “no *DKK1”* TCGA-LIHC tumors. Left panel: Volcano plot showing gene-wise changes (log2 fold change versus statistical significance). Right panel: Enrichment analysis of genes upregulated in *DKK1*-high tumors using TRANScription FACtor database and JASPAR position weight matrices (TRANSFAC and JASPAR PWMs). Analysis reveals a robust enrichment of TEAD-dependent transcriptional signatures, with TEAD2 and TEAD4 identified as the top-ranking motifs. **(C)** UMAP of all cells from the scRNA-seq dataset (GEO; accession GSE149614), colored by cell-type annotation. Major lineages are indicated: hepatocyte, EC, LSEC, fibroblast, myeloid, T, B, NK, plasma, and mast cells. **(D)** UMAP feature plots showing expression of canonical lineage markers used for cell-type assignment; The expression levels of the indicated genes across UMAP clusters are visualized as a heatmap on the right; panel frame colors correspond to the lineage color scheme in (C). **(E)** Dot plot summarizing canonical marker expression across annotated cell types; color denotes log2 fold change (cell type versus all other cells) and dot size denotes the fraction of cells expressing each marker. **(F, G)** scRNA-seq analyses (GEO; accession GSE149614) showing that *DKK1* mRNA expression is predominantly enriched in hepatocytes, whereas YAP target genes are upregulated in hepatocytes and multiple surrounding stromal populations. (F) UMAPs showing expression of Hippo-YAP/TAZ-TEAD axis genes: *DKK1; YAP1* and canonical YAP targets (*ANKRD1, CTGF*, and *CYR61*); *LRP6*; and *TEAD2* and *TEAD4* (YAP/TAZ binding partner TFs); The expression levels of the indicated genes across UMAP clusters are visualized as a heatmap on the right. (G) Heatmap of log2 fold change (cell type versus all others) for the indicated genes; red = higher expression, blue = lower expression. Statistical analysis was performed using an unpaired t-test. Exact *P* values are shown in the figures; *P* < 0.05 was considered statistically significant. Abbreviations: *ALB,* albumin; *ANKRD1*, ankyrin repeat domain 1; *APOA2*, apolipoprotein A2; *AXIN1*, axis inhibition protein 1; B, B cell; *CD3D*, CD3 delta subunit of T-cell receptor complex; *CD3E*, CD3 epsilon subunit of T-cell receptor complex; *CD3G*, CD3 gamma subunit of T-cell receptor complex; *CD68*, CD68 molecule; *CD79A*, CD79a molecule; *CLEC4G*, c-type lectin domain family 4 member G; *COL1A1*, collagen type I alpha 1 chain; *CPA3*, carboxypeptidase A3; *CRHBP*, corticotropin releasing hormone binding protein; *CTGF,* connective tissue growth factor; *CTNNB1*, catenin beta 1; *CYR61*, cysteine-rich angiogenic inducer 61; *DCN*, decorin; *DKK1*, dickkopf-1; EC, endothelial cells; *EMCN*, endomucin; *FCN3*, ficolin *3*; GEO, gene expression omnibus; GSE, GEO series; HCC, hepatocellular carcinoma; *HNF4A*, hepatocyte nuclear factor 4 alpha; *KLRD1*, killer cell lectin like receptor D1; *KLRF1*, killer cell lectin like receptor F1; LIHC, liver hepatocellular carcinoma; *LRP6*, low-density lipoprotein receptor-related protein 6; LSEC, liver sinusoidal endothelial cells; *LYZ*, lysozyme; *MS4A1*, membrane spanning 4-domains A1; mut, mutant; *MZB1*, marginal zone B and B1 cell specific protein; NK, natural killer cell; *PECAM1*, platelet and endothelial cell adhesion molecule 1; scRNA-seq, single-cell RNA-sequencing; *SERPINA1*, serpin family A member 1; T, T cell; TCGA, The Cancer Genome Atlas; *TEAD*, transcriptional enhanced associate domain; TF, transcription factor; *TPSAB1*, tryptase alpha/beta 1; *TPSB2*, tryptase beta 2; *TP53*, tumor protein P53; TRANSFAC and JASPAR PWMs, transcription factor database and JASPAR position weight matrices; UMAP, Uniform Manifold Approximation and Projection; WT, wild-type; *YAP1*, yes associated protein 1.

**Supplementary Figure S2. DKK1 acts as a putative upstream regulator of YAP target gene expression in hepatocellular carcinoma.**

DKK1 expression is associated with YAP/TEAD activity in HCC and pharmacologic DKK1 inhibition suppresses Hep3B xenograft growth. **(A)** Immunohistochemical analysis of an HCC tissue microarray (*n* = 67) was used to evaluate YAP, DKK1, and CTGF protein expression in tumor versus normal liver cores. Normal liver cores showed negative or weak staining for all three markers. IHC scores were defined as follows: 0, no staining or faint staining in < 30% of cells; 1+, faint staining in ≥ 30% of cells; 2+, weak to moderate staining in ≥ 30% of cells; and 3+, strong staining in ≥ 30% of cells. Cases with scores of 2+ or 3+ were classified as the positive expression group. In HCC cores, YAP1, DKK1, and CTGF showed positive expression in 29/67 (43.3%), 18/67 (26.9%), and 29/67 (43.3%) cases, respectively. Among YAP-positive tumors (*n* = 29), 21/29 (72.4%) and 15/29 (51.7%) also displayed CTGF and DKK1 positive expression, respectively. Bottom, correlation analysis of IHC scores (0-3+) for DKK1, YAP1, and CTGF across individual HCC cases (*n* = 67); associations were assessed using Spearman’s rank correlation (ρ), with corresponding *P* values shown on each plot (all correlations were significant; *P* < 0.001). **(B)** Using the DepMap database (https://depmap.org/portal/), we compared *DKK1* gene expression levels across multiple HCC cell lines and found that Hep3B exhibited the highest *DKK1* mRNA level, with Huh7 and HepG2 ranking 6^th^ and 19^th^ among 27 HCC cell lines, respectively. **(C)** qPCR analysis of *DKK1* mRNA levels in the human embryonic kidney cell line HEK293T and HCC cell lines (HepG2, Huh7, Hep3B). Expression was normalized to HEK293T. *DKK1* mRNA was markedly elevated in HCC cell lines and was highest in Hep3B. **(D)** Immunoblotting analysis of the human embryonic kidney cell line HEK293T, the non-tumor liver cell line MIHA, and HCC cell lines (HepG2, Huh7, Hep3B). DKK1 protein was highest in Hep3B. Relative to MIHA, HCC cell lines exhibited reduced LATS1 phosphorylation; HepG2 showed increased total YAP, whereas Huh7 and Hep3B exhibited low YAP phosphorylation. **(E)** Endpoint tumor images and tumor weights from Hep3B xenograft-bearing mice treated orally with vehicle or the DKK1 inhibitor WAY-262611 (16 mg/kg in 200 μL, once daily for 10 days), showing reduced tumor size and weight with WAY-262611. **(F)** WAY-262611 treatment reduced tumor volume over time in Hep3B xenograft models compared with the vehicle (DMSO) - treated group. **(G)** qPCR analysis of YAP target genes (*CTGF* and *CYR61*) in excised Hep3B xenograft tumors, showing reduced mRNA expression following WAY-262611 treatment. **(H)** Estimation of DKK1 concentration in DKK1-CM by DKK1 immunoprecipitation followed by immunoblotting. Specifically, rhDKK1 0, 25, 50, 100, 200, 400 and 800 ng/mL was included as a standard series to generate a band-intensity standard curve; the DKK1-CM signal was converted to concentration using the standard-curve equation, yielding an estimated DKK1 concentration of around 1,962.21 ng/mL. **(I)** qPCR analysis revealed that treatment with DKK1-CM upregulated YAP target gene expression and modestly decreased expression of the Wnt target gene *AXIN2* in Huh7 cells. Unless otherwise indicated, statistical analyses were performed using one-way ANOVA; panels (E-G) were analyzed using an unpaired t-test, and correlations in (A) were assessed by Spearman’s rank correlation. Exact *P* values are shown in the figure; *P* < 0.05 was considered statistically significant. Data in panels (C, D, E, I) are presented as mean ± SD, and data in panels (F) are presented as mean ± SEM. Abbreviations: ANOVA, analysis of variance; *AXIN2*, axis inhibition protein 2; *CTGF,* connective tissue growth factor; *CYR61*, cysteine-rich angiogenic inducer 61; *DKK1*, dickkopf-1; DKK1-CM, DKK1-conditioned medium; HCC, hepatocellular carcinoma; qPCR, quantitative PCR; rhDKK1, recombinant human DKK1; SD, standard deviation; SEM, standard error of the mean; YAP, yes-associated protein.

**Supplementary Figure S3. DKK1 promotes hepatocellular carcinoma progression in a YAP/TAZ-dependent manner.**

Transwell migration/invasion and wound-healing assays were used to test whether DKK1-driven pro-migratory and pro-invasive phenotypes in HCC cells require YAP/TAZ-TEAD activity. **(A, B)** Transwell migration and invasion assays were performed in Huh7 cells treated with Ctrl-CM or DKK1-CM, with or without *siYAP + siTAZ*. DKK1-CM increased migration and invasion, whereas *siYAP + siTAZ* attenuated the DKK1-CM-induced increases. Migration and invasion were quantified as the stained area relative to the total area (*n* = 3) (A). Knockdown efficiency was confirmed by qPCR (*n* = 3) (B). **(C, D)** Huh7 cells were treated with MGH-CP1 (10 µmol/L), a TEAD auto palmitoylation inhibitor that disrupts YAP/TAZ-TEAD complex formation and TEAD-dependent transcription, and analyzed by Transwell migration and invasion assays under Ctrl-CM or DKK1-CM conditions. MGH-CP1 treatment suppressed DKK1-CM-induced migration and invasion. Migration and invasion were quantified as the stained area relative to the total area (*n* = 3) (C). Inhibition of TEAD-dependent transcription was verified by reduced *CTGF* and *CYR61* mRNA expression measured by qPCR (*n* = 3) (D). **(E)** Wound-healing assays were performed in Huh7 cells treated with Ctrl-CM or DKK1-CM, with or without *siYAP + siTAZ*. Wound closure was quantified from images acquired at 0 and 24 h by measuring the remaining wound distance using ImageJ (*n* = 3). **(F)** YAP-TEAD transcriptional activity in Huh7 cells following Flag-DKK1 overexpression was measured using an 8×GTIIC dual-luciferase reporter assay (*n* = 3). Unless otherwise indicated, statistical analyses were performed using one-way ANOVA; panel (F) was analyzed using an unpaired t-test. Exact *P* values are shown in the figure; *P* < 0.05 was considered statistically significant. Data are presented as mean ± SD. Abbreviations: ANOVA, analysis of variance; Ctrl-CM, control-conditioned medium; *CTGF*, connective tissue growth factor; *CYR61*, cysteine-rich angiogenic inducer 61; DKK1, dickkopf-1; DKK1-CM, DKK1-conditioned medium; HCC, hepatocellular carcinoma; qPCR, quantitative PCR; SD, standard deviation; TAZ, transcriptional coactivator with PDZ-binding motif; TEAD, transcriptional enhanced associate domain transcription factor; YAP, yes-associated protein.

**Supplementary Figure S4. DKK1 suppresses Hippo signaling, leading to YAP activation.**

Molecular and biochemical assays were performed to define the mechanism by which DKK1 modulates YAP/TEAD-dependent gene expression. **(A)** PLA in Hep3B cells showing reduced TEAD1-YAP interaction upon DKK1 knockdown. Red puncta represent amplified PLA signals indicating close proximity of TEAD1 and YAP (i.e., interaction events). PLA signals were quantified as dots per cell (*n* = 20 cells). **(B)** qPCR analysis showing that DKK1 knockdown does not alter *YAP* mRNA expression in Hep3B cells (*n* = 3). **(C)** qPCR analysis showing that DKK1-CM does not change *YAP* or *TAZ* mRNA levels in Huh7 cells (*n* = 3). **(D)** Western blotting analysis demonstrating reduced YAP protein levels following DKK1 knockdown in Hep3B cells. DKK1 and YAP protein levels were quantified as the ratio of each protein’s intensity to β-actin intensity (*n* = 3). **(E)** Generation of DKK1-KO Hep3B cell lines using CRISPR-Cas9 with three independent *sgRNAs* targeting DKK1; To generate DKK1-KO Hep3B cells, we cloned each *sgDKK1* into the lentiCRISPRv2 vector and transfected HEK293T cells to produce CRISPR/Cas9 lentivirus targeting DKK1. The lentiviral supernatant was collected and used to infect Hep3B cells, and DKK1-KO clones were obtained through antibiotic selection followed by cell sorting; loss of DKK1 protein was confirmed by Western blotting. Knockout control cells were generated with a *non-targeting sgRNA.* **(F)** Western blotting analysis showing reduced YAP protein levels in Hep3B DKK1-KO cells. **(G)** Nucleus/cytosol fractionation analysis showed that DKK1-CM treatment increased nuclear localization of YAP, whereas nuclear β-catenin levels remained unchanged. **(H)** Immunofluorescence analysis in high-confluency Hep3B cultures showing that DKK1 knockdown promotes cytosolic redistribution of YAP. The nuclear-to-cytoplasmic YAP ratio was quantified based on fluorescence intensity (*n* = 20 cells). **(I)** Western blotting analysis showing that DKK1-CM treatment decreases p-YAP S127 in Huh7 cells. YAP phosphorylation was quantified as the p-YAP/YAP band-intensity ratio (*n* = 3). **(J)** Western blotting demonstrated that DKK1 knockdown with *siDKK1* elevated p-LATS1 levels, and this effect was rescued by rhDKK1 (200 ng/mL) treatment. **(K)** To assess upstream Hippo kinase activity, cells overexpressing EGFP-MST1 were treated with DKK1-CM and analyzed for MST1 and LATS1 phosphorylation by immunoblotting. DKK1-CM did not detectably change MST1 phosphorylation but reduced LATS1 phosphorylation, consistent with decreased Hippo pathway activity. Statistical analysis was performed using one-way ANOVA unless otherwise indicated. For panel (G, I), an unpaired t-test was used. Exact *P* values are shown in the figures; *P* < 0.05 was considered statistically significant. Figure panels (A-D) and (H, I) are presented as mean ± SD. Abbreviations: ANOVA, analysis of variance; Ctrl-CM, control-conditioned medium; DKK1, dickkopf-1; DKK1-KO, dickkopf-1 knockout cell lines; DKK1-CM, DKK1-conditioned medium; KO, knock-out; LATS1, large tumor suppressor kinase 1; MST1, mammalian STE20-like protein kinase 1; PLA, proximity ligation assay; qPCR, quantitative PCR; SD, standard deviation; *sgRNA*, single guide RNA; TEAD, transcriptional enhanced associate domain transcription factor; YAP, yes-associated protein.

**Supplementary Figure S5. DKK1 inhibits the interaction between Merlin and LATS1, thereby reducing membrane localization of LATS1.**

DKK1 disrupts LATS1 membrane localization and LATS1-Merlin complex formation in HCC cells. **(A)** Membrane fractionation analysis in Hep3B DKK1-KO cells treated with DKK1-CM showed reduced membrane-associated LATS1. Na⁺/K⁺- ATPase and β-tubulin were used as markers for the membrane fraction and total lysates, respectively. **(B)** Immunofluorescence analysis in Huh7 cells stimulated with rhDKK1 showed decreased membrane co-localization of LATS1 and Merlin. White arrows indicate regions where the LATS1 and Merlin signals are colocalized at the membrane. **(C)** Co-immunoprecipitation of endogenous proteins demonstrated that DKK1-CM treatment reduced the interaction between LATS1 and Merlin. **(D)** PLA in Hep3B cells showed that treatment with the DKK1 inhibitor WAY-262611 increased LATS1-Merlin proximity/interaction. Red puncta indicate amplified PLA signals corresponding to LATS1-Merlin interaction events. PLA signals were quantified as dots per cell (*n* = 20 cells). Statistical analysis was performed using one-way ANOVA. Exact *P* values are shown in the figures; *P* < 0.05 was considered statistically significant. Data in panel (D) are presented as mean ± SD. Abbreviations: ANOVA, analysis of variance; Ctrl-CM, control-conditioned medium; DKK1, dickkopf-1; DKK1-CM, DKK1-conditioned medium; DKK1-KO, dickkopf-1 knockout cell lines; HCC, hepatocellular carcinoma; KO, knock-out; LATS1, large tumor suppressor kinase 1; Merlin, moesin-ezrin-radixin-like protein; PLA, proximity ligation assay; rhDKK1, recombinant human DKK1; SD, standard deviation; WCL, whole cell lysate.

**Supplementary Figure S6. DKK1 promotes YAP activation in an LRP6-dependent manner that occurs independently of canonical Wnt signaling.**

The contribution of canonical Wnt signaling and the Wnt co-receptor LRP6 to DKK1-mediated Hippo pathway regulation was examined using Wnt and YAP/TEAD reporter assays, immunofluorescence, and immunoblotting. **(A)** Canonical Wnt/β-catenin signaling was measured in HEK293T cells using the pSuperTOP (TCF/LEF) dual-luciferase reporter. Wnt3a-CM induced reporter activity, and co-treatment with DKK1-CM inhibited this induction. **(B)** As in (A), Wnt3a-CM-induced pSuperTOP activity was inhibited by co-treatment with SOST1-CM, indicating effective blockade of canonical Wnt signaling by an LRP6-binding Wnt antagonist. **(C)** YAP subcellular localization was assessed by immunofluorescence in high-confluency Huh7 cells treated with DKK1-CM or SOST1-CM. DKK1-CM, but not SOST1-CM, promoted YAP nuclear localization. The nuclear-to-cytoplasmic YAP ratio was quantified by fluorescence intensity (*n* = 20 cells). **(D)** Schematic of LRP6 WT and the LRP6 m5 mutant, in which the serine/threonine residues within all five PPPS/TP motifs are substituted with alanine. **(E)** Reporter assays in HEK293T cells overexpressing VSVG-tagged LRP6 WT or VSVG-tagged LRP6 m5. VSVG-LRP6 WT overexpression increased pSuperTOP activity, whereas VSVG-LRP6 6m5 did not; in contrast, both LRP6 WT and LRP6 m5 overexpression increased YAP-TEAD reporter activity (8×GTIIC dual-luciferase) (*n* = 3). **(F)** Reporter assays in Huh7 cells overexpressing VSVG-LRP6 WT or VSVG-LRP6 m5. As in (E), VSVG-LRP6 WT overexpression, but not VSVG-LRP6 m5, activated the pSuperTOP reporter, while both constructs increased 8×GTIIC YAP-TEAD reporter activity (*n* = 3). **(G)** Immunofluorescence analysis of Huh7 cells treated with rhDKK1 (200 ng/mL) showing increased nuclear YAP without an obvious change in β-catenin localization. **(H)** Immunofluorescence analysis of Huh7 LRP6-KO cells reconstituted with VSVG-LRP6 WT or VSVG-LRP6 m5 shows that either construct promotes YAP nuclear localization without an apparent change in β-catenin localization. **(I)** Quantification of (G): YAP nuclear-to-cytoplasmic ratio determined from fluorescence intensity measurements (*n* = 20 cells). **(J)** Quantification of (H): YAP nuclear-to-cytoplasmic ratio determined from fluorescence intensity measurements (*n* = 20 cells). **(K)** Western blotting analysis in Huh7 cells showing that VSVG-LRP6 m5 overexpression caused a modest reduction in active (non-phosphorylated) β-catenin (detected using an antibody against unphosphorylated β-catenin) together with reduced YAP phosphorylation. **(L)** Western blotting analysis showing that β-catenin knockdown (*siCTNNB1*) did not alter YAP phosphorylation in Huh7 cells. For panels (I) and (J), an unpaired t-test was used. Exact *P* values are shown in the figures; *P* < 0.05 was considered statistically significant. Figure panels (A-B), (E-F), (I-J) are presented as mean ± SD. Abbreviations: Ctrl-CM, control-conditioned medium; DKK1, dickkopf-1; LRP6, low-density lipoprotein receptor-related protein 6; LRP6-KO, low-density lipoprotein receptor-related protein 6 knockout cell lines; CM, conditioned medium; SD, standard deviation; SOST, sclerostin; YAP, yes-associated protein; TEAD, transcriptional enhanced associate domain transcription factor; rhDKK1, recombinant human DKK1; KO, knockout.

**Supplementary Figure S7. LRP6-dependent coupling of DKK1 to YAP pathway output and DKK1-driven hepatocellular carcinoma cell motility.**

**(A)** TIMER2.0 analysis (https://compbio.cn/timer2/) of TCGA-LIHC patient samples showing correlations between *DKK1* and representative YAP/TEAD target genes (as indicated). Samples were stratified by *LRP6* mRNA expression into *LRP6*-High (top 30%) and *LRP6*-Low (bottom 30%) groups, revealing that the strength of the DKK1-YAP target gene correlations depends on *LRP6* expression level. **(B)** Schematic of the ROTAC-Ctrl construct and ROTACs engineered to target LRP5/6, including the LRP6-directed design (ROTAC-LRP6). **(C)** Western blotting analysis in Huh7 cells showing that ROTAC-LRP6 overexpression decreases LRP6 abundance and, concomitantly, reduces total YAP protein levels. **(D)** Western blotting analysis in Hep3B cells showing that ROTAC-LRP6 overexpression decreases LRP6 abundance and increases YAP phosphorylation. **(E)** Transwell migration and invasion assays in Huh7 cells treated with Ctrl-CM or DKK1-CM, with or without ROTAC-LRP6 overexpression. DKK1-CM increased migration and invasion compared with Ctrl-CM, whereas ROTAC-LRP6 overexpression markedly attenuated these DKK1-CM-induced effects. Migration and invasion were quantified as the stained area relative to the total area (*n* = 3). Statistical analysis was performed using one-way ANOVA. Exact *P* values are shown in the figures; *P* < 0.05 was considered statistically significant. Figure panel (E) is presented as mean ± SD. Abbreviations: ANOVA, analysis of variance; Ctrl-CM, control-conditioned medium; DKK1, dickkopf-1; DKK1-CM, DKK1-conditioned medium; LIHC, liver hepatocellular carcinoma; LRP6, low-density lipoprotein receptor-related protein 6; ROTAC, R-spondin targeting chimera; ROTAC-LRP6, LRP6 targeting ROTAC; SD, standard deviation; YAP, yes-associated protein.

**Supplementary Figure S8. DKK1 induces *O*-GlcNAcylation of LRP6 and suppresses the Hippo signaling pathway.**

Biochemical assays were performed to define how DKK1 regulates LRP6 *O*-GlcNAcylation, LRP6-OGT complex formation, and Hippo pathway activity. **(A)** In Hep3B cells, DKK1 knockdown decreased LRP6 *O*-GlcNAcylation as assessed by sWGA pull-down, which enriches *O*-GlcNAc-modified proteins via terminal GlcNAc binding. Specificity of protein *O*-GlcNAcylation was confirmed by free GlcNAc competition, which inhibits bead binding to *O*-GlcNAcylated proteins. **(B)** In Huh7 cells, DKK1-EGFP overexpression 48h after transfection increased LRP6 *O*-GlcNAcylation, and the DKK1-EGFP overexpression-induced increase in LRP6 *O*-GlcNAcylation was attenuated by treatment with the DKK1 inhibitor WAY-262611 (4 µmol/L), as determined by sWGA pull-down followed by immunoblotting for LRP6. **(C, D)** PLA were used to detect endogenous LRP6-OGT proximity/interaction in Huh7 (C) and Hep3B (D) cells. PLA puncta were quantified as dots per cell (*n* = 20 cells). **(E)** PLA analysis in Huh7 cells detecting LRP6-Merlin proximity/interaction; PLA puncta were quantified as dots per cell (*n* = 20 cells). **(F)** Treatment of Hep3B DKK1-KO cells with rhDKK1 (200 ng/mL) increased LRP6-OGT proximity/interaction as measured by PLA. **(G)** Quantification of (F): PLA puncta per cell were counted using ImageJ (*n* = 20 cells). **(H)** To test canonical Wnt signaling dependence, LRP6 WT or the Wnt-signaling-defective LRP6 m5 mutant (shown in Supplementary Figure S6D) was immunoprecipitated and analyzed for *O*-GlcNAcylation. Flag-OGT overexpression induced comparable *O*-GlcNAcylation of LRP6 WT and LRP6 m5, indicating that LRP6 *O*-GlcNAcylation does not require intact PPPS/TP motif-mediated Wnt signaling. **(I)** sWGA pull-down analysis comparing the effects of Flag-DKK1 versus SOST1 overexpression 48 h after transfection showed that Flag-DKK1 overexpression, but not SOST1 overexpression, induces LRP6 *O*-GlcNAcylation. **(J)** Under serum-deprived conditions that favor Hippo pathway activation, DKK1-CM increased LRP6 *O*-GlcNAcylation and reduced LATS1 phosphorylation, consistent with Hippo pathway suppression. Statistical analysis was performed using one-way ANOVA. Exact *P* values are shown in the figures; *P* < 0.05 was considered statistically significant. Figure panels are presented as mean ± SD. Abbreviations: ANOVA, analysis of variance; CM, conditioned medium; DKK1, dickkopf-1; DKK1-KO, dickkopf-1 knockout cell lines; KO, knockout; LATS1, large tumor suppressor kinase 1; LRP6, low-density lipoprotein receptor-related protein 6; LRP6-WT, LRP6 wild-type; Merlin, moesin-ezrin-radixin-like protein; OGT, *O*-GlcNAc transferase; PLA, proximity ligation assay; rhDKK1, recombinant human DKK1; SD, standard deviation; SOST, sclerostin; sWGA, succinylated wheat germ agglutinin; WCL, whole cell lysate; WT, wild-type.

**Supplementary Figure S9. DKK1-CM-induced temporal changes in LRP6-Merlin and LATS-Merlin interactions.**

A time-course was performed in Hep3B DKK1-KO cells treated with DKK1-CM to define the temporal order of events linking DKK1 to the OGT-LRP6 axis and Hippo pathway regulation. DKK1-CM increased OGT membrane localization and LRP6 *O*-GlcNAcylation within 5 min, increased LRP6-Merlin association from 15 min, and reduced LATS1-Merlin association at later time points (30-60 min). **(A)** Membrane fractionation followed by immunoblotting showed that OGT membrane localization increased within 5 min of DKK1-CM treatment and rose to 5.72-fold above baseline by 30 min. Na⁺/K⁺-ATPase and β-tubulin were used as markers for the membrane fraction and cytosolic fraction, respectively. **(B)** LRP6 *O*-GlcNAcylation was assessed by sWGA bead pull-down at 0, 5, 10, 15, and 30 min after DKK1-CM treatment. LRP6 *O*-GlcNAcylation increased within 5 min and remained elevated through 30 min. **(C)** PLA analysis of LRP6-Merlin proximity/interaction in Hep3B DKK1-KO cells after DKK1-CM treatment showed increased PLA puncta at 15-30 min. PLA signals were quantified as dots per cell (*n* = 20 cells). **(D)** PLA analysis of LATS1-Merlin proximity/interaction in Hep3B DKK1-KO cells after DKK1-CM treatment showed decreased PLA puncta at 30-60 min. PLA signals were quantified as dots per cell (*n* = 20 cells). Statistical analysis was performed using one-way ANOVA. Exact *P* values are shown in the figures; *P* < 0.05 was considered statistically significant. Figure panels (C) and (D) are presented as mean ± SD. Abbreviations: ANOVA, analysis of variance; CM, conditioned medium; DKK1, dickkopf-1; DKK1-KO, dickkopf-1 knockout cell lines; KO, knockout; LATS1, large tumor suppressor kinase 1; LRP6, low-density lipoprotein receptor-related protein 6; Merlin, moesin-ezrin-radixin-like protein; OGT, *O*-GlcNAc transferase; PLA, proximity ligation assay; SD, standard deviation; sWGA, succinylated wheat germ agglutinin; WCL, whole cell lysate.

**Supplementary Figure S10. The region near the PPPS/TP motif A supports the LRP6-OGT interaction, thereby inhibiting the Hippo signaling pathway.**

A domain mapping analysis was conducted to determine which domain of LRP6 is regulated by DKK1. **(A)** Schematic of LRP6 WT and the indicated deletion constructs. **(B)** IP analysis following Flag-OGT overexpression showed *O*-GlcNAcylation of LRP6 WT and LRP6 ΔN-term, whereas *O*-GlcNAcylation was not detected on the LRP6 ΔC-term, indicating that the cytoplasmic tail is required for modification. **(C)** IP analysis demonstrated that OGT associated with LRP6 WT and LRP6 ΔN-term, but not with LRP6 ΔC-term, indicating that the LRP6 cytoplasmic region is necessary for OGT binding. **(D)** sWGA pull-down analysis showed that DKK1-CM induced *O*-GlcNAcylation of LRP6 WT, whereas LRP6 ΔC-term failed to undergo DKK1-CM-induced *O*-GlcNAcylation. **(E)** Schematic diagram of a series of LRP6 deletion mutant constructs. **(F)** IP analysis under Flag-OGT overexpression showed that *O*-GlcNAcylation of LRP6 was largely abolished in the ΔABCDE mutant, whereas it was markedly restored in the ΔBCDE mutant, narrowing the major regulatory region to surrounding the motif A. **(G)** Upper, schematic of the LRP6 ΔA construct (deletion of residues 1487-1526). Lower, IP analysis showing that deletion of the A region markedly reduced LRP6 *O*-GlcNAcylation. **(H)** IP analysis under Flag-OGT overexpression showed that LRP6 ΔA exhibited reduced binding to OGT and to Merlin compared with wild-type LRP6. **(I)** Generation of HEK293T LRP6-KO clones; To generate HEK293T LRP6-KO cells, we cloned each *sgLRP6* into the lentiCRISPRv2 vector and transfected HEK293T cells to produce CRISPR/Cas9 lentivirus targeting LRP6. The lentiviral supernatant was collected and used to infect HEK293T cells, and LRP6-KO clones were obtained through antibiotic selection followed by cell sorting. Knockout efficiency was assessed by Western blotting. Knockout control cells were generated with a *non-targeting sgRNA*. **(J)** Effect of LRP6 loss on Hippo signaling assessed by immunoblotting quantification of LATS1 phosphorylation. **(K)** qPCR analysis showing reduced expression of YAP/TEAD target genes in HEK293T LRP6-KO cells (*n* = 3). **(L)** In HEK293T LRP6-KO cells reconstituted with wild-type LRP6 or LRP6 ΔA, Western blotting analysis of LATS1 and YAP phosphorylation showed that LRP6 ΔA suppresses Hippo signaling less effectively than wild-type LRP6. Statistical analysis was performed using one-way ANOVA. Exact *P* values are shown in the figures; *P* < 0.05 was considered statistically significant. Figure panel (K) is presented as mean ± SD. Abbreviations: ANOVA, analysis of variance; DKK1, dickkopf-1; DKK1-CM, DKK1-conditioned medium; IP, immunoprecipitation; LATS1, large tumor suppressor kinase 1; LRP6, low-density lipoprotein receptor-related protein 6; LRP6-KO, low-density lipoprotein receptor-related protein 6 knockout cell lines; OGT, *O*-GlcNAc transferase; qPCR, quantitative PCR; SD, standard deviation; sWGA, succinylated wheat germ agglutinin; YAP, yes-associated protein; WCL, whole cell lysate; ΔN-term, N-terminal deleted mutant; ΔC-term, C-terminal-deleted mutant.

**Supplementary Figure S11. DKK1 inhibits the Hippo signaling pathway by inducing *O*-GlcNAcylation at the T1466 residue of LRP6.**

Mass spectrometry and functional analyses of site-directed mutants were used to define LRP6 *O*-GlcNAcylation sites and to determine their contribution to Hippo/YAP signaling output. **(A)** Representative tandem mass spectra of HexNAc-modified LRP6 peptides (AHVTGASSSSSSSTK, HFAPPTTPCSTDVCDSDYAPSRR, and SQYLSAEENYEScPPSPYTER) with annotated fragment ions confirming site-specific *O*-GlcNAc modification. Modified serine/threonine residues are shown in bold; carbamidomethylated cysteine is indicate1d as bold lowercase (c). XCorr scores were 8.33, 5.92, and 2.83, respectively (Supplementary Table S1). **(B)** In silico prediction of *O*-GlcNAcylation likelihood at candidate residues (T1466, S1533, and S1575) using the deep learning-based tool O-GlcNAcPRED-DL (<http://oglcnac.org/pred_dl>). **(C)** Co-immunoprecipitation analysis of Flag-Merlin with LRP6 variants showing that the LRP6 T1466A mutation, but not LRP6 S1533A, reduces Merlin binding. **(D)** YAP-TEAD transcriptional activity was measured after overexpression of LRP6 WT or the indicated alanine-substitution mutants (T1466A, S1533A, S1575A) using an 8×GTIIC luciferase reporter in HEK293T LRP6-KO cells. The LRP6 T1466A mutation attenuated LRP6 overexpression-driven TEAD activation, whereas LRP6 S1533A and LRP6 S1575A induced reporter activity comparable to LRP6 WT. **(E)** Generation of Huh7 LRP6-KO cell lines using CRISPR-Cas9 with three independent *single guide RNAs (#1, #2 and #3).* Loss of LRP6 protein was confirmed by Western blotting. Knockout control cells were generated with a *non-targeting sgRNA*. **(F)** In Huh7 LRP6-KO cells, co-immunoprecipitation analysis of endogenous Merlin-LATS1 complexes following reconstitution with LRP6 WT or LRP6 T1466A overexpression showed that LRP6 WT reduced Merlin-LATS1 interaction, whereas the LRP6 T1466A mutant did not. **(G)** CHX (100 μg/mL) followed by Western blotting revealed reduced protein stability of LRP6 T1466A compared with LRP6 WT. **(H)** S100/P100-based membrane fractionation of Huh7 LRP6-KO cells overexpressing LRP6 WT or LRP6 T1466A showed no appreciable difference in membrane-associated LRP6 between constructs. Na⁺/K⁺-ATPase and β-tubulin were used as markers/loading controls for membrane and cytosolic fractions, respectively. Statistical analysis was performed using one-way ANOVA. Exact *P* values are shown in the figures; *P* < 0.05 was considered statistically significant. Figure panel (D) is presented as mean ± SD. Abbreviations: ANOVA, analysis of variance; CHX, cycloheximide; DKK1, dickkopf-1; DKK1-CM, DKK1-conditioned medium; sgRNA, single guide-RNA; LATS1, large tumor suppressor kinase 1; LRP6, low-density lipoprotein receptor-related protein 6; LRP6-KO, low-density lipoprotein receptor-related protein 6 knockout cell lines; Merlin, moesin-ezrin-radixin-like protein; OGT, *O*-GlcNAc transferase; SD, standard deviation; TEAD, transcriptional enhanced associate domain transcription factor; WCL, whole cell lysate; YAP, yes-associated protein.

**Supplementary Figure S12. DKK1-mediated PIP_3_ formation induces the PIP_3_-OGT interaction and LRP6 *O*-GlcNAcylation.**

Evaluation of DKK1 domain requirements and PIP_3_ dependency in DKK1-induced LRP6 *O*-GlcNAcylation. **(A)** Schematic of DKK1 WT and DKK1 ΔCRD1 or DKK1 ΔCRD2. **(B)** CM collected from HEK293T cells overexpressing DKK1 WT, DKK1 ΔCRD1, or DKK1 ΔCRD2 were immunoprecipitated with a DKK1-specific antibody and immunoblotted to confirm the presence of each secreted DKK1 variant in CM. **(C)** sWGA bead pull-down (enrichment of *O*-GlcNAc-modified proteins) followed by immunoblotting for LRP6 showed that DKK1-CM induced LRP6 *O*-GlcNAcylation after 15 min, whereas CM containing DKK1 ΔCRD1 or DKK1 ΔCRD2 failed to induce LRP6 *O*-GlcNAcylation. **(D)** High-confluency Huh7 cells were serum-starved for 1 h and treated with the indicated CM, followed by immunofluorescence analysis of YAP localization. DKK1-CM promoted YAP nuclear accumulation, whereas DKK1 ΔCRD1-CM and DKK1 ΔCRD2-CM did not. YAP nuclear-to-cytoplasmic ratio was quantified by fluorescence intensity (*n* = 20 cells). **(E)** PLA was used to assess OGT-PIP_3_ proximity/interaction in Huh7 cells. **(F)** Quantification of (E): PLA puncta per cell were counted using ImageJ (*n* = 20 cells). **(G)** PLA analysis in Hep3B cells showed that the OGT-PIP_3_ interaction was reduced by wortmannin (PI3K inhibitor). PLA signals were quantified as puncta per cell using ImageJ (*n* = 20 cells). **(H-J)** LRP6 *O*-GlcNAcylation was analyzed by sWGA pull-down under the indicated PIP_3_-promoting conditions. Enrichment specificity was confirmed by free GlcNAc competition, which blocks sWGA binding to *O*-GlcNAc–modified proteins. **(H)** Overexpression of constitutively active EGFP-PIK3CA E545K, which promotes PIP_3_ production, increased LRP6 *O*-GlcNAcylation. **(I)** LRP6 *O*-GlcNAcylation was increased upon overexpression of Myr-EGFP-PIK3CB, a membrane-localized construct that induces PIP_3_ production**. (J)** LRP6 *O*-GlcNAcylation was increased upon *siPTEN*, a condition that leads to intracellular PIP_3_ accumulation. **(K)** Immunoprecipitation analysis showed that *siPTEN* (to increase PIP_3_) enhanced the endogenous interaction between LRP6 and Merlin. Arrow denotes the band at the expected molecular weight of Merlin. Statistical analysis was performed using one-way ANOVA. Exact *P* values are shown in the figures; *P* < 0.05 was considered statistically significant. Figure panels (D), (F) are presented as mean ± SD. Abbreviations: ANOVA, analysis of variance; Ctrl-CM, control-conditioned medium; CRD, cysteine rich domain; CM, conditioned medium; DKK1, dickkopf-1; DKK1ΔCRD1, deletion mutants DKK1 lacking cysteine-rich domain 1; DKK1ΔCRD2, deletion mutants DKK1 lacking cysteine-rich domain 2; LRP6, low-density lipoprotein receptor-related protein 6; Merlin, moesin-ezrin-radixin-like protein; PTEN, phosphatase and tensin homolog; PLA, proximity ligation assay; PIP_3_, phosphatidylinositol (3,4,5)-trisphosphate; SD, standard deviation; sWGA, succinylated wheat germ agglutinin; WCL, whole cell lysate, YAP, yes-associated protein.

**Supplementary Figure S13. DKK1-induced PIP_3_ formation recruits OGT to the membrane, thereby inducing LRP6 *O*-GlcNAcylation.**

OGT subcellular localization and the functional consequences of membrane-localized OGT on LRP6 *O*-GlcNAcylation and Hippo pathway activity were examined. **(A)** HEK293T cells were treated with rhDKK1 (200 ng/mL) for 15 min, and LRP6 *O*-GlcNAcylation was assessed by sWGA bead pull-down followed by immunoblotting. Addition of rhDKK1 markedly increased LRP6 *O*-GlcNAcylation, without appreciable changes in OGT *O*-GlcNAcylation, global protein *O*-GlcNAcylation, or total OGT abundance in WCL. **(B)** Immunofluorescence analysis showed increased membrane-associated OGT signal following DKK1-CM treatment. White arrows indicate areas with increased intensity of membrane-localized OGT. **(C)** Membrane-localized OGT in (B) was quantified as relative membrane OGT fluorescence intensity (*n* = 4). **(D)** Membrane fractionation followed by Western blotting confirmed increased OGT in membrane fractions after DKK1-CM treatment. Na⁺/K⁺-ATPase and β-tubulin were used as markers/loading controls for membrane and cytosolic fractions, respectively. **(E)** Schematic of the generation of the membrane-targeted OGT fusion construct (TmEGFP-OGT), in which the signal peptide domain and transmembrane domain of LRP6 ΔN-term construct were fused to the N-terminus of EGFP-OGT. **(F)** Western blotting analysis of membrane fractions showing that TmEGFP-OGT exhibits enhanced membrane localization compared with EGFP-OGT. **(G)** Under serum-free (Hippo-active) conditions, TmEGFP-OGT suppressed Hippo signaling more effectively than EGFP-OGT, as indicated by reduced phosphorylation of LATS1 and YAP by immunoblotting. **(H)** Immunoprecipitation analysis comparing LRP6 *O*-GlcNAcylation showed that TmEGFP-OGT overexpression promotes LRP6 *O*-GlcNAcylation more efficiently than EGFP-OGT overexpression. **(I)** Schematic of EGFP-OGT, the PPO-deletion mutant (EGFP-OGT ΔPPO), and the membrane-targeted PPO-deletion fusion (TmEGFP-OGT ΔPPO). **(J)** Membrane fractionation analysis showing that, despite deletion of the PPO domain, membrane targeting (TmEGFP-OGT ΔPPO) increases OGT membrane localization relative to EGFP-OGT ΔPPO. Statistical analysis was performed using unpaired t test, and exact *P* values are shown in each figure; *P* < 0.05, statistically significant. Figure panel (C) is presented as mean ± SD. Abbreviations: DKK1, dickkopf-1; PIP_3_, phosphatidylinositol (3,4,5)-trisphosphate; OGT, *O*-GlcNAc transferase; LRP6, low-density lipoprotein receptor-related protein 6; rhDKK1, recombinant human DKK1; CM, conditioned medium; SD, standard deviation; sWGA, succinylated wheat germ agglutinin; WCL, whole cell lysate; PPO, phosphoinositide-binding-domain.

**Supplementary Figure S14. Overexpression of EGFP-PPO inhibits migration and invasion of hepatocellular carcinoma cell lines.**

The anti-cancer potential and efficacy of a PPO-domain construct were assessed. **(A)** Conceptual model illustrating a PPO construct-based therapeutic strategy in which PPO acts as a PIP_3_-binding “mask” to limit PIP_3_-dependent OGT membrane recruitment, thereby reducing LRP6 *O*-GlcNAcylation and downstream YAP/TEAD-driven tumor phenotypes in DKK1-overexpressing or PIP_3_-upregulated cancers. **(B)** PLA detecting proximity/interaction between EGFP-PPO and PIP_3_ (*n* = 11). Statistical analysis was performed using one-way ANOVA. Exact *P* values are shown in the figures; *P* < 0.05 was considered statistically significant. **(C)** Transwell migration and invasion assays in Hep3B cells showing that EGFP-PPO overexpression reduces migratory and invasive capacity. Migration and invasion were quantified as stained area relative to total area. Statistical analysis was performed using unpaired t test, and exact *P* values are shown in each figure; *P* < 0.05, statistically significant. Figure panel (B, C) is presented as mean ± SD. Abbreviations: ANOVA, analysis of variance; DKK1, dickkopf-1; OGT, *O*-GlcNAc transferase; PIP_3_, phosphatidylinositol (3,4,5)-trisphosphate; PLA, proximity ligation assay; PPO, phosphoinositide-binding domain of OGT; SD, standard deviation.

**Supplementary Figure S15. PPO overexpression acts as a competitive inhibitor, specifically suppressing YAP activity in tumor cells.**

Tumor-selective Hippo/YAP regulation and the anti-cancer potential of PPO overexpression were evaluated in non-tumor cells, DKK1-high HCC cells, and PTEN-deficient (PIP_3_-high) cancer cells, and compared with global pharmacologic inhibition of OGT. **(A)** Cells were treated with OSMI-1 (30 µmol/L, 16 h), a small-molecule OGT inhibitor, and analyzed by immunoblotting. OSMI-1 decreased YAP protein levels and reduced global protein *O*-GlcNAcylation in HEK293, Huh7, and Hep3B cells. **(B)** Immunoblotting analysis showing that EGFP-PPO overexpression in Huh7 cells decreases YAP protein levels. **(C)** In PTEN-deficient U87-MG and PC-3 cells (PIP_3_-high), EGFP-PPO overexpression reduced YAP protein levels while causing only minimal changes in total protein *O*-GlcNAcylation, in contrast to OSMI-1, which broadly decreased global *O*-GlcNAcylation. **(D)** Schematic of the lentiviral TetON inducible PPO overexpression vector. **(E)** Doxycycline induction of PPO overexpression was assessed by immunoblotting in Hep3B and HEK293 cells. PPO induction in DKK1-high Hep3B cells markedly reduced YAP protein levels without appreciably altering total *O*-GlcNAcylation; in contrast, induced PPO overexpression produced no detectable change in YAP or total *O*-GlcNAcylation in HEK293 cells. Abbreviations: DKK1, dickkopf-1; OGT, *O*-GlcNAc transferase; PIP_3_, phosphatidylinositol (3,4,5)-trisphosphate; PPO, phosphoinositide-binding domain of OGT; PTEN, phosphatase and tensin homolog; YAP, yes-associated protein.

**Supplementary Figure S16. Early oncogenic mutation-induced DKK1 expression promotes HCC progression via LRP6 *O*-GlcNAcylation and Hippo pathway suppression.**

(I) Acquisition of an early oncogenic driver mutation initiates HCC. (II) Oncogenic signaling promotes tumor cell proliferation and expansion. (III) As the tumor mass increases, mechanical constraints and cell-cell contact activate Hippo signaling and contact inhibition, limiting further proliferation by restraining YAP activity. (IV) HCC-derived DKK1 creates a niche that suppresses the Hippo pathway, thereby abolishing contact inhibition and supporting continuous proliferation. Proposed molecular mechanism: DKK1 induces PIP_3_ production, which recruits OGT to the plasma membrane and promotes LRP6 *O*-GlcNAcylation, culminating in inhibition of Hippo signaling and sustained YAP activation. (V) Persistent YAP activation drives uncontrolled proliferation and tumor progression. Abbreviations: DKK1, dickkopf-1; HCC, hepatocellular carcinoma; LATS1, large tumor suppressor kinase 1; LRP6, low-density lipoprotein receptor-related protein 6; Merlin, moesin-ezrin-radixin-like protein; OGT, *O*-GlcNAc transferase; PIP_3,_ phosphatidylinositol (3,4,5)-trisphosphate_;_ YAP, yes-associated protein.
