# Supplementary material for: DKK1 Suppresses Hippo Signaling via PIP3–OGT–LRP6 O-GlcNAcylation in Hepatocellular Carcinoma
Source: Cancer Commun (Lond). 2026 Apr 27;46:0022. doi: 10.34133/cancomm.0022 (PMC13112786; doi:10.34133/cancomm.0022)
Supplement: Supplementary 1 — Materials and Methods Tables S1 to S5 Figs. S1 to S16 [file cancomm.0022.f1.zip › Supplementary Figure S1.pdf]

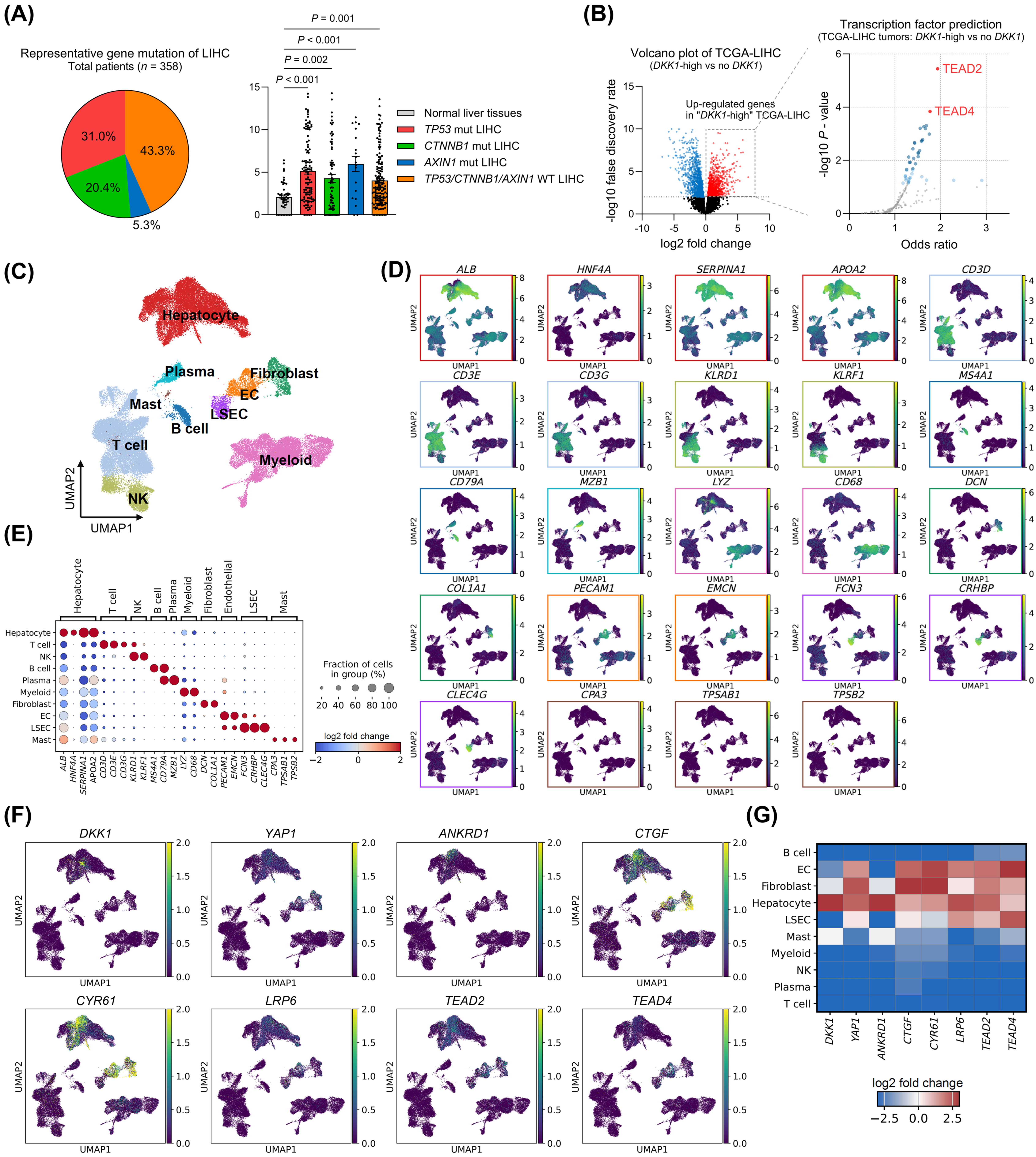
(A)
Representative gene mutation of LIHC
Total patients (n = 358)
31.0%
43.3%
20.4%
5.3%
P = 0.001
P < 0.001
P = 0.002
P < 0.001
Normal liver tissues
TP53 mut LIHC
CTNNB1 mut LIHC
AXIN1 mut LIHC
TP53/CTNNB1/AXIN1 WT LIHC
(B)
Volcano plot of TCGA-LIHC
(DKK1-high vs no DKK1)
Up-regulated genes
in "DKK1-high" TCGA-LIHC
-log10 false discovery rate
log2 fold change
Transcription factor prediction
(TCGA-LIHC tumors: DKK1-high vs no DKK1)
TEAD2
TEAD4
-log10 P - value
Odds ratio
(C)
Hepatocyte
Plasma
Fibroblast
EC
Mast
LSEC
B cell
T cell
Myeloid
NK
UMAP2
UMAP1
(D)
ALB
HNF4A
SERPINA1
APOA2
CD3D
CD3E
CD3G
KLRD1
KLRF1
MS4A1
CD79A
MZB1
LYZ
CD68
DCN
COL1A1
PECAM1
EMCN
FCN3
CRHBP
CLEC4G
CPA3
TPSAB1
TPSB2
(E)
Hepatocyte
T cell
NK
B cell
Plasma
Myeloid
Fibroblast
Endothelial
LSEC
Mast
EC
Fraction of cells
in group (%)
20 40 60 80 100
log2 fold change
(F)
DKK1
YAP1
ANKRD1
CTGF
CYR61
LRP6
TEAD2
TEAD4
(G)
log2 fold change
