# Supplementary material for: DKK1 Suppresses Hippo Signaling via PIP3–OGT–LRP6 O-GlcNAcylation in Hepatocellular Carcinoma
Source: Cancer Commun (Lond). 2026 Apr 27;46:0022. doi: 10.34133/cancomm.0022 (PMC13112786; doi:10.34133/cancomm.0022)
Supplement: Supplementary 1 — Materials and Methods Tables S1 to S5 Figs. S1 to S16 [file cancomm.0022.f1.zip › Supplementary Figure S10.pdf]

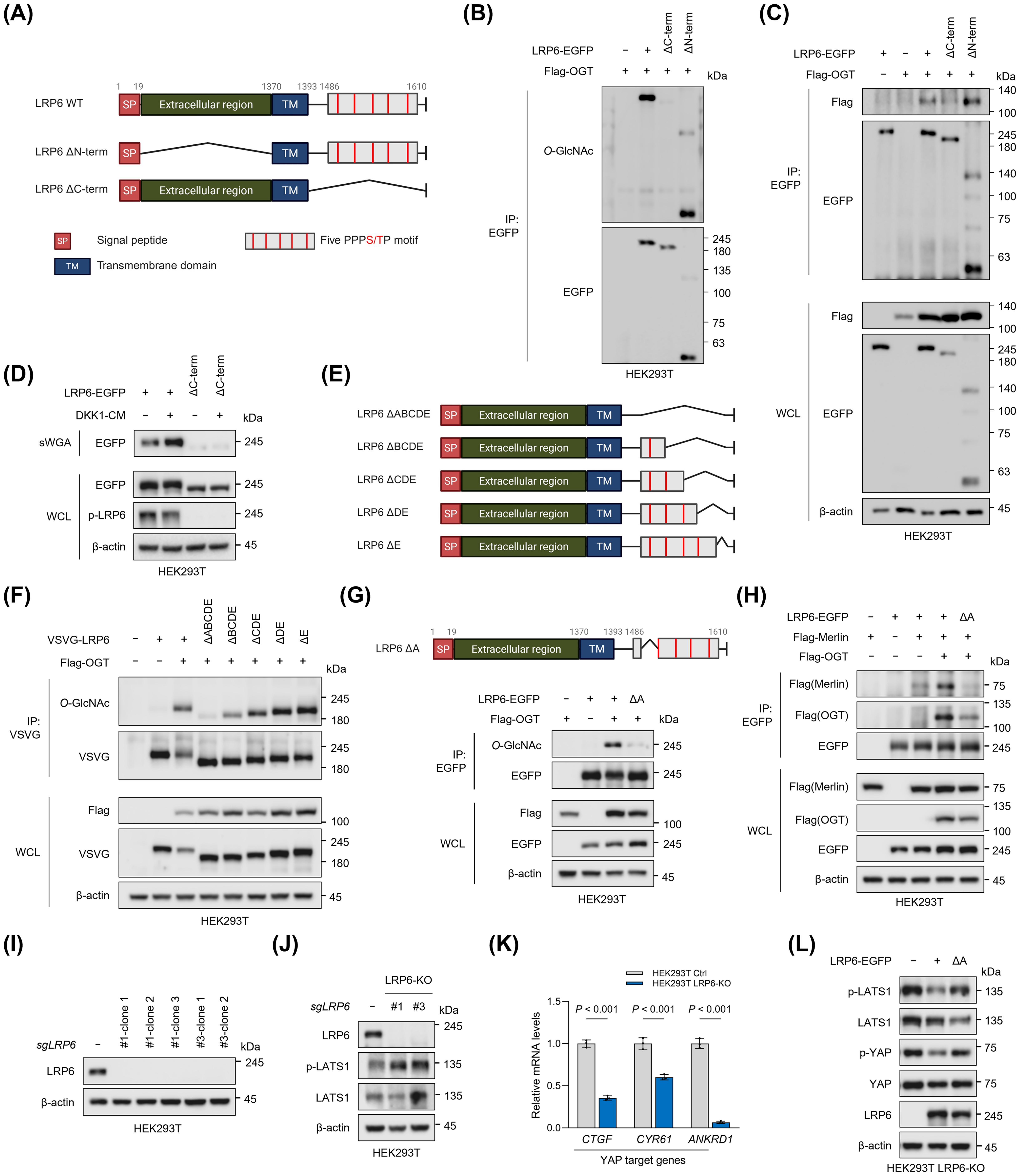

(A)
LRP6 WT
1 19 1370 1393 1486 1610
SP
Extracellular region
TM
LRP6 ΔN-term
LRP6 ΔC-term
Signal peptide
Transmembrane domain
Five PPPS/TP motif
(B)
LRP6-EGFP − + ΔC-term ΔN-term
Flag-OGT + + + +
kDa
O-GlcNAc
EGFP
IP: EGFP
245 180 135 100 75 63
HEK293T
(C)
LRP6-EGFP + − + ΔC-term ΔN-term
Flag-OGT − + + + +
Flag
EGFP
IP: EGFP
WCL
β-actin
140 100 245 180 140 100 75 63 45
HEK293T
(D)
LRP6-EGFP + + ΔC-term ΔC-term
DKK1-CM − + − +
sWGA
EGFP
WCL
p-LRP6
β-actin
245 45
HEK293T
(E)
LRP6 ΔABCDE
LRP6 ΔBCDE
LRP6 ΔCDE
LRP6 ΔDE
LRP6 ΔE
(F)
VSVG-LRP6 − + + ΔABCDE ΔBCDE ΔCDE ΔDE ΔE
Flag-OGT − − + + + + + +
O-GlcNAc
VSVG
IP: VSVG
Flag
WCL
β-actin
245 180 100 45
HEK293T
(G)
LRP6 ΔA
1 19 1370 1393 1486 1610
LRP6-EGFP − + + ΔA
Flag-OGT + − + +
O-GlcNAc
EGFP
IP: EGFP
Flag
WCL
β-actin
245 100 45
HEK293T
(H)
LRP6-EGFP − + + + ΔA
Flag-Merlin + − + + +
Flag-OGT − − − + +
Flag(Merlin)
Flag(OGT)
EGFP
IP: EGFP
WCL
β-actin
75 135 100 245 45
HEK293T
(I)
sgLRP6 − #1-clone 1 #1-clone 2 #1-clone 3 #3-clone 1 #3-clone 2
LRP6
β-actin
245 45
HEK293T
(J)
LRP6-KO
sgLRP6 − #1 #3
LRP6
p-LATS1
LATS1
β-actin
245 135 45
HEK293T
(K)
HEK293T Ctrl
HEK293T LRP6-KO
Relative mRNA levels
0.0 0.5 1.0 1.5
P < 0.001
CTGF CYR61 ANKRD1
YAP target genes
(L)
LRP6-EGFP − + ΔA
p-LATS1
LATS1
p-YAP
YAP
LRP6
β-actin
135 75 245 45
HEK293T LRP6-KO
