# Supplementary material for: DKK1 Suppresses Hippo Signaling via PIP3–OGT–LRP6 O-GlcNAcylation in Hepatocellular Carcinoma
Source: Cancer Commun (Lond). 2026 Apr 27;46:0022. doi: 10.34133/cancomm.0022 (PMC13112786; doi:10.34133/cancomm.0022)
Supplement: Supplementary 1 — Materials and Methods Tables S1 to S5 Figs. S1 to S16 [file cancomm.0022.f1.zip › Supplementary Figure S12.pdf]

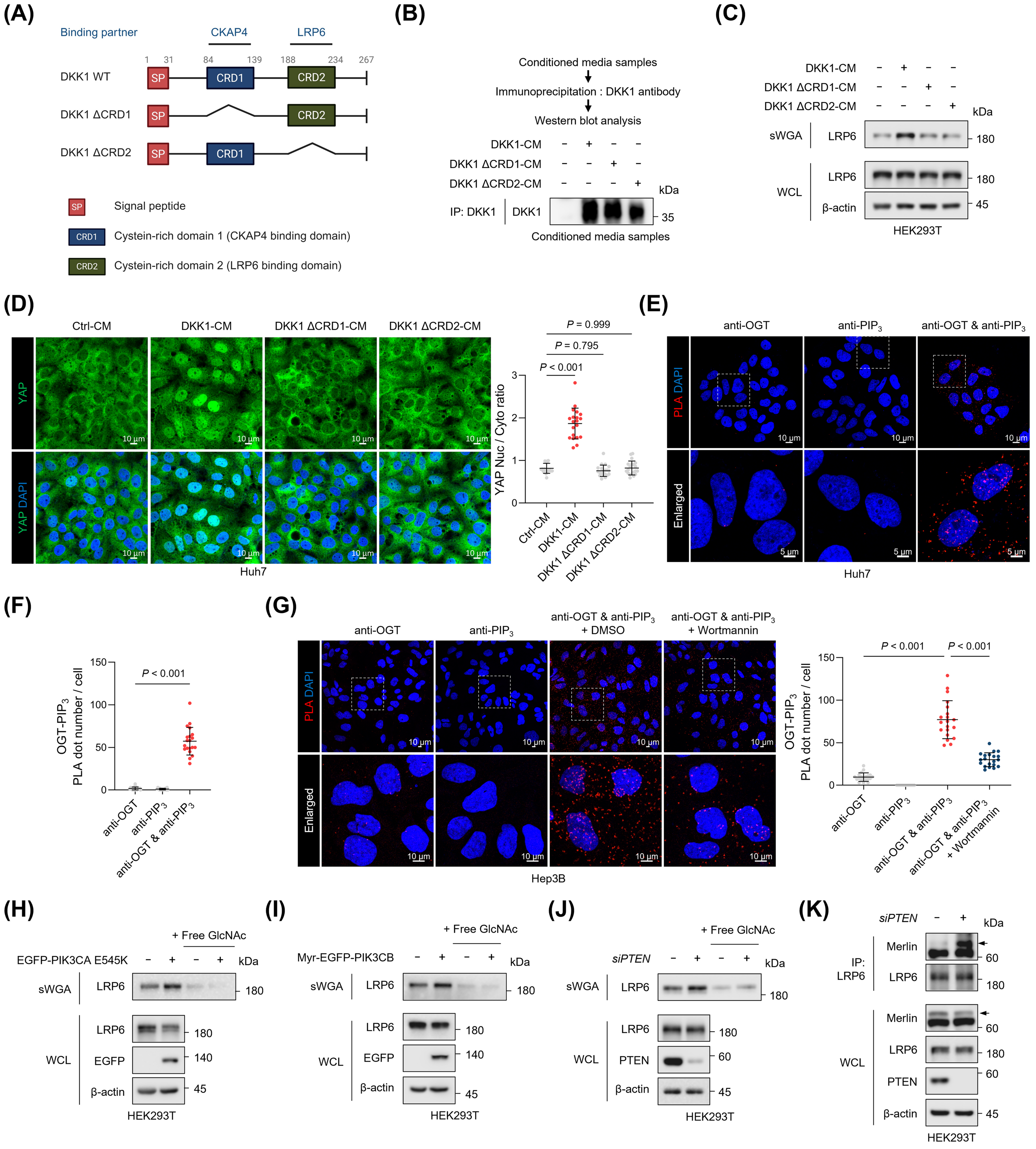

(A)
Binding partner
CKAP4
LRP6
DKK1 WT
DKK1 ΔCRD1
DKK1 ΔCRD2
SP
CRD1
CRD2
Signal peptide
Cystein-rich domain 1 (CKAP4 binding domain)
Cystein-rich domain 2 (LRP6 binding domain)
(B)
Conditioned media samples
Immunoprecipitation : DKK1 antibody
Western blot analysis
DKK1-CM
DKK1 ΔCRD1-CM
DKK1 ΔCRD2-CM
IP: DKK1
DKK1
kDa
Conditioned media samples
(C)
sWGA
WCL
LRP6
β-actin
HEK293T
(D)
Ctrl-CM
DKK1-CM
DKK1 ΔCRD1-CM
DKK1 ΔCRD2-CM
YAP
YAP DAPI
Huh7
YAP Nuc / Cyto ratio
P = 0.999
P = 0.795
P < 0.001
(E)
anti-OGT
anti-PIP3
anti-OGT & anti-PIP3
PLA DAPI
Enlarged
Huh7
(F)
OGT-PIP3 PLA dot number / cell
P < 0.001
(G)
anti-OGT & anti-PIP3 + DMSO
anti-OGT & anti-PIP3 + Wortmannin
Hep3B
(H)
+ Free GlcNAc
EGFP-PIK3CA E545K
EGFP
(I)
Myr-EGFP-PIK3CB
(J)
siPTEN
PTEN
(K)
IP: LRP6
Merlin
