# Supplementary material for: DKK1 Suppresses Hippo Signaling via PIP3–OGT–LRP6 O-GlcNAcylation in Hepatocellular Carcinoma
Source: Cancer Commun (Lond). 2026 Apr 27;46:0022. doi: 10.34133/cancomm.0022 (PMC13112786; doi:10.34133/cancomm.0022)
Supplement: Supplementary 1 — Materials and Methods Tables S1 to S5 Figs. S1 to S16 [file cancomm.0022.f1.zip › Supplementary Figure S13.pdf]

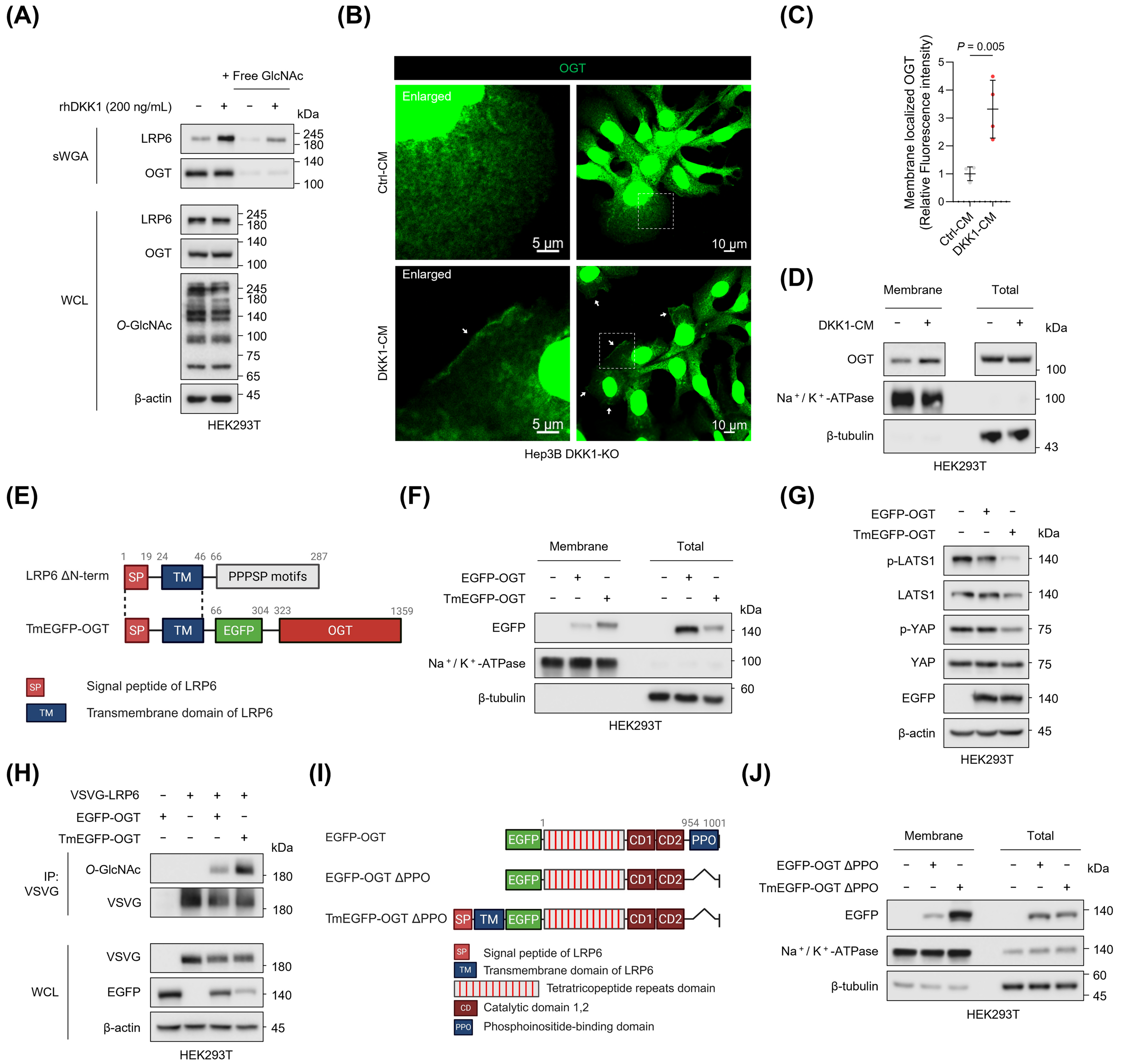

(A)
+ Free GlcNAc
rhDKK1 (200 ng/mL)
kDa
sWGA
LRP6
OGT
WCL
O-GlcNAc
β-actin
HEK293T
(B)
OGT
Enlarged
Ctrl-CM
DKK1-CM
5 μm
10 μm
Hep3B DKK1-KO
(C)
P = 0.005
Membrane localized OGT (Relative Fluorescence intensity)
Ctrl-CM
DKK1-CM
(D)
Membrane
Total
DKK1-CM
OGT
Na⁺/K⁺-ATPase
β-tubulin
HEK293T
(E)
LRP6 ΔN-term
TmEGFP-OGT
SP
TM
PPPSP motifs
EGFP
OGT
Signal peptide of LRP6
Transmembrane domain of LRP6
(F)
EGFP-OGT
TmEGFP-OGT
EGFP
(G)
p-LATS1
LATS1
p-YAP
YAP
(H)
VSVG-LRP6
IP: VSVG
VSVG
(I)
EGFP-OGT ΔPPO
TmEGFP-OGT ΔPPO
CD1
CD2
PPO
Tetratricopeptide repeats domain
Catalytic domain 1,2
Phosphoinositide-binding domain
(J)
EGFP-OGT ΔPPO
TmEGFP-OGT ΔPPO
