# Supplementary material for: DKK1 Suppresses Hippo Signaling via PIP3–OGT–LRP6 O-GlcNAcylation in Hepatocellular Carcinoma
Source: Cancer Commun (Lond). 2026 Apr 27;46:0022. doi: 10.34133/cancomm.0022 (PMC13112786; doi:10.34133/cancomm.0022)
Supplement: Supplementary 1 — Materials and Methods Tables S1 to S5 Figs. S1 to S16 [file cancomm.0022.f1.zip › Supplementary Figure S14.pdf]

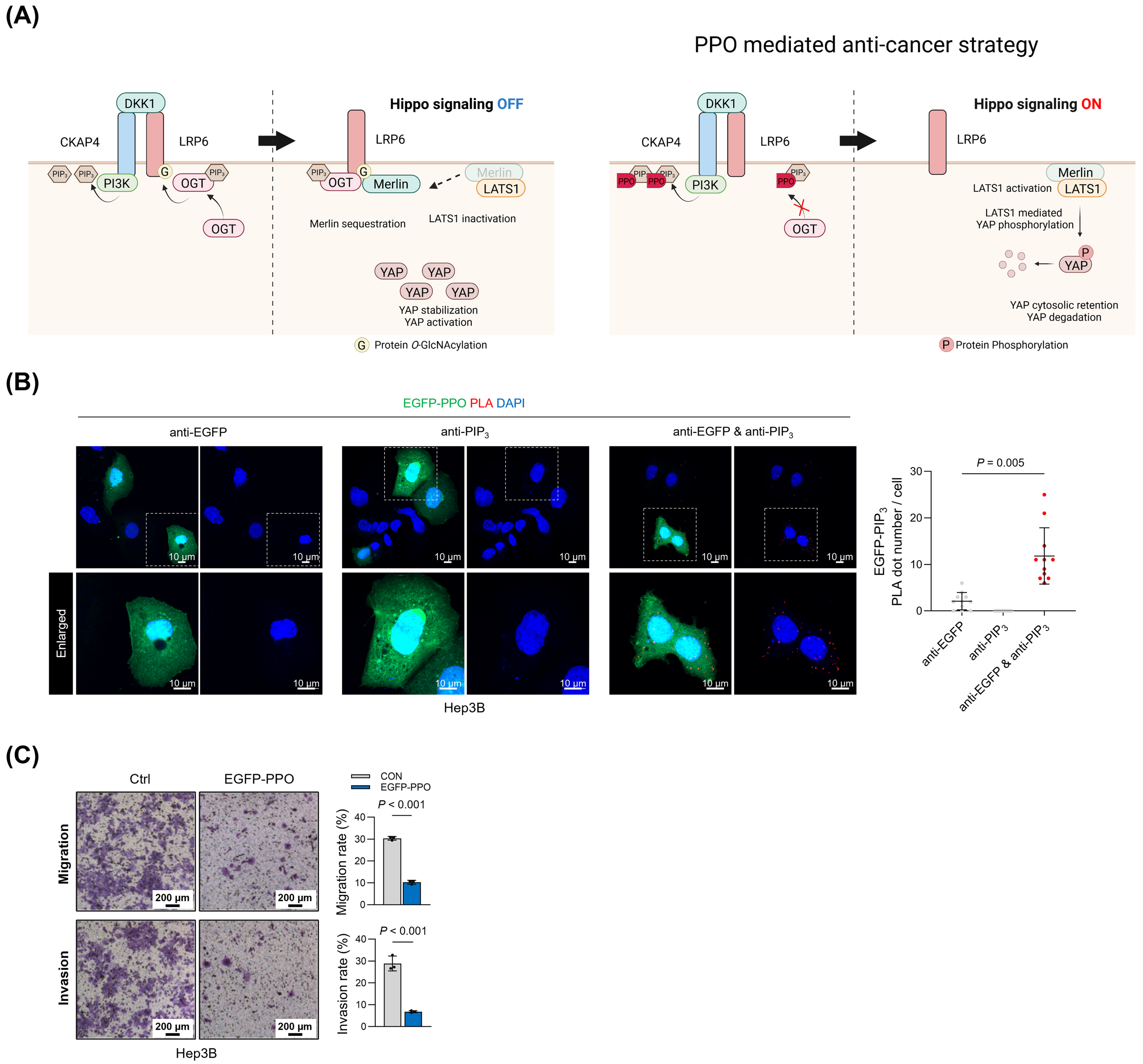

(A)
PPO mediated anti-cancer strategy
DKK1
CKAP4
LRP6
PI3K
OGT
Hippo signaling OFF
Merlin
LATS1
Merlin sequestration
LATS1 inactivation
YAP
YAP stabilization
YAP activation
Protein O-GlcNAcylation
Hippo signaling ON
LATS1 activation
LATS1 mediated
YAP phosphorylation
YAP cytosolic retention
YAP degadation
Protein Phosphorylation
(B)
EGFP-PPO PLA DAPI
anti-EGFP
anti-PIP3
anti-EGFP & anti-PIP3
Enlarged
10 μm
Hep3B
P = 0.005
EGFP-PIP3
PLA dot number / cell
(C)
Ctrl
EGFP-PPO
Migration
Invasion
200 μm
Hep3B
CON
EGFP-PPO
P < 0.001
Migration rate (%)
Invasion rate (%)
