# Supplementary material for: DKK1 Suppresses Hippo Signaling via PIP3–OGT–LRP6 O-GlcNAcylation in Hepatocellular Carcinoma
Source: Cancer Commun (Lond). 2026 Apr 27;46:0022. doi: 10.34133/cancomm.0022 (PMC13112786; doi:10.34133/cancomm.0022)
Supplement: Supplementary 1 — Materials and Methods Tables S1 to S5 Figs. S1 to S16 [file cancomm.0022.f1.zip › Supplementary Figure S15.pdf]

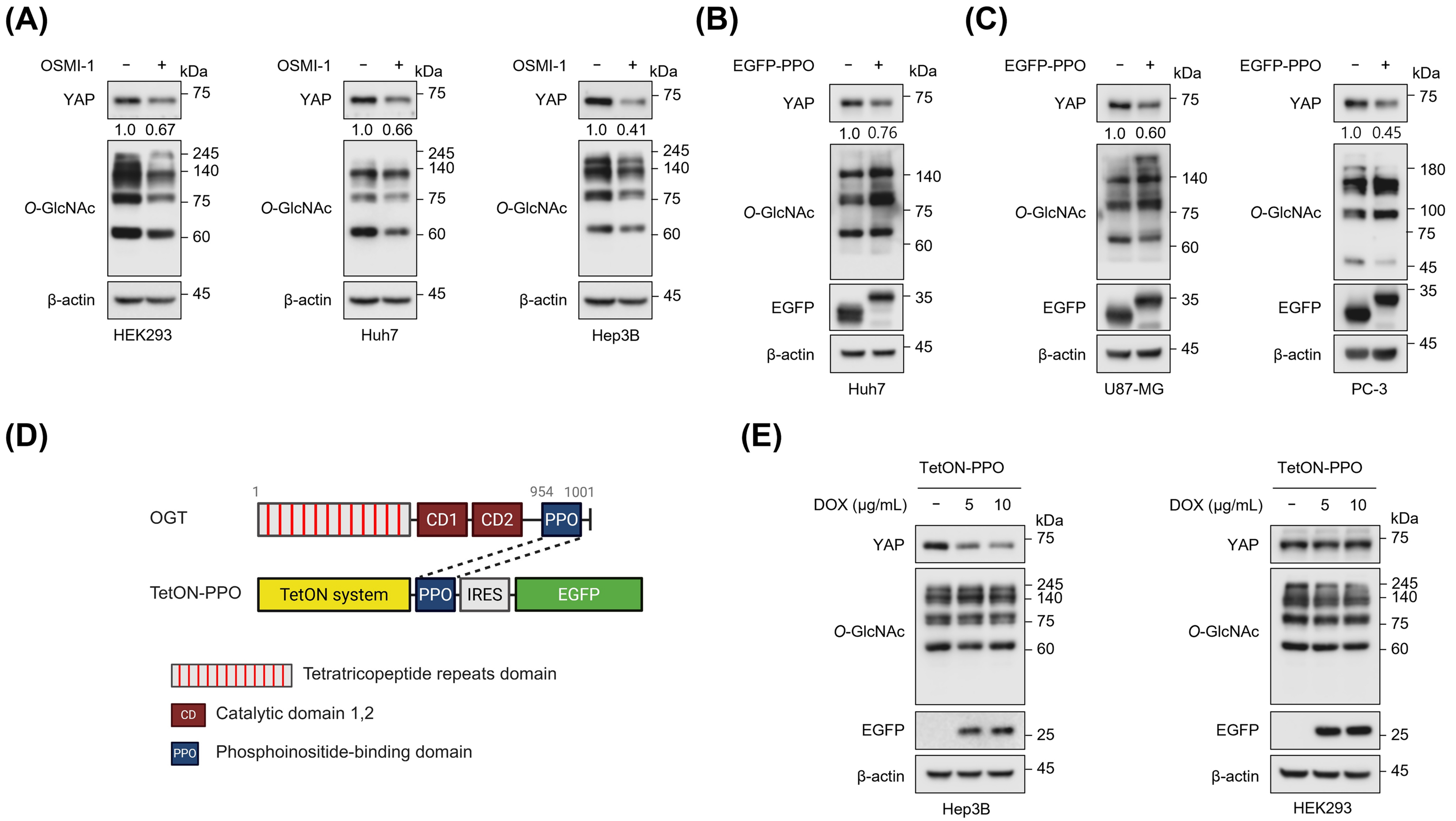

(A)
OSMI-1 – + kDa
YAP 75
1.0 0.67
O-GlcNAc 245 140 75 60
β-actin 45
HEK293
OSMI-1 – + kDa
YAP 75
1.0 0.66
O-GlcNAc 245 140 75 60
β-actin 45
Huh7
OSMI-1 – + kDa
YAP 75
1.0 0.41
O-GlcNAc 245 140 75 60
β-actin 45
Hep3B
(B)
EGFP-PPO – + kDa
YAP 75
1.0 0.76
O-GlcNAc 140 75 60
EGFP 35
β-actin 45
Huh7
(C)
EGFP-PPO – + kDa
YAP 75
1.0 0.60
O-GlcNAc 140 75 60
EGFP 35
β-actin 45
U87-MG
EGFP-PPO – + kDa
YAP 75
1.0 0.45
O-GlcNAc 180 100 75 45
EGFP 35
β-actin 45
PC-3
(D)
1 954 1001
OGT
CD1 CD2 PPO
TetON-PPO
TetON system PPO IRES EGFP
Tetratricopeptide repeats domain
CD Catalytic domain 1,2
PPO Phosphoinositide-binding domain
(E)
TetON-PPO
DOX (μg/mL) – 5 10
kDa
YAP 75
O-GlcNAc 245 140 75 60
EGFP 25
β-actin 45
Hep3B
TetON-PPO
DOX (μg/mL) – 5 10
kDa
YAP 75
O-GlcNAc 245 140 75 60
EGFP 25
β-actin 45
HEK293
