# Supplementary material for: DKK1 Suppresses Hippo Signaling via PIP3–OGT–LRP6 O-GlcNAcylation in Hepatocellular Carcinoma
Source: Cancer Commun (Lond). 2026 Apr 27;46:0022. doi: 10.34133/cancomm.0022 (PMC13112786; doi:10.34133/cancomm.0022)
Supplement: Supplementary 1 — Materials and Methods Tables S1 to S5 Figs. S1 to S16 [file cancomm.0022.f1.zip › Supplementary Figure S16.pdf]

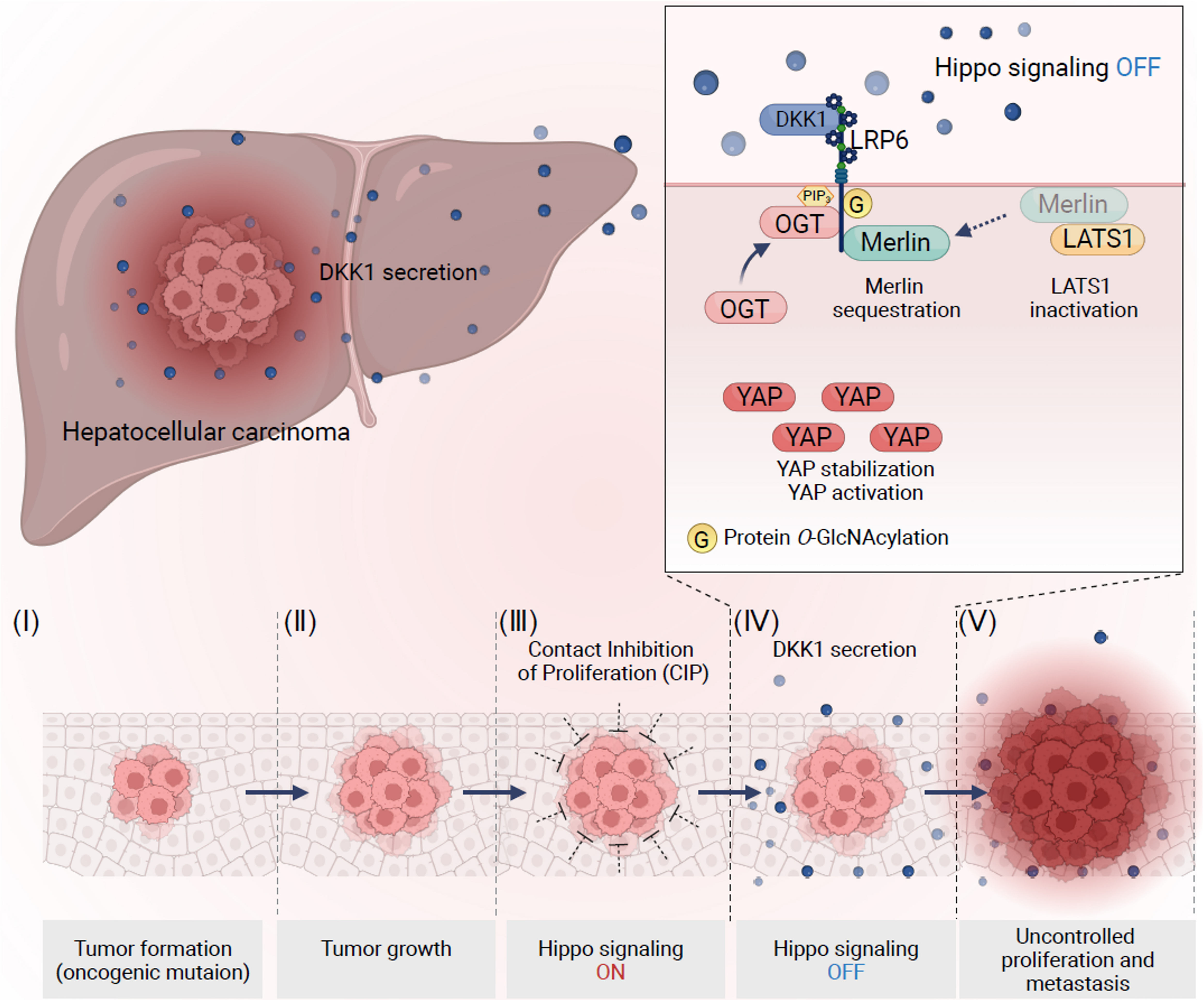
Hippo signaling OFF
DKK1
LRP6
PIP3
G
OGT
Merlin
Merlin
LATS1
OGT
Merlin sequestration
LATS1 inactivation
YAP
YAP
YAP
YAP
YAP stabilization
YAP activation
G Protein O-GlcNAcylation
DKK1 secretion
Hepatocellular carcinoma
(I)
(II)
(III)
Contact Inhibition of Proliferation (CIP)
(IV)
DKK1 secretion
(V)
Tumor formation (oncogenic mutaion)
Tumor growth
Hippo signaling ON
Hippo signaling OFF
Uncontrolled proliferation and metastasis
