# Supplementary material for: DKK1 Suppresses Hippo Signaling via PIP3–OGT–LRP6 O-GlcNAcylation in Hepatocellular Carcinoma
Source: Cancer Commun (Lond). 2026 Apr 27;46:0022. doi: 10.34133/cancomm.0022 (PMC13112786; doi:10.34133/cancomm.0022)
Supplement: Supplementary 1 — Materials and Methods Tables S1 to S5 Figs. S1 to S16 [file cancomm.0022.f1.zip › Supplementary Figure S2.pdf]

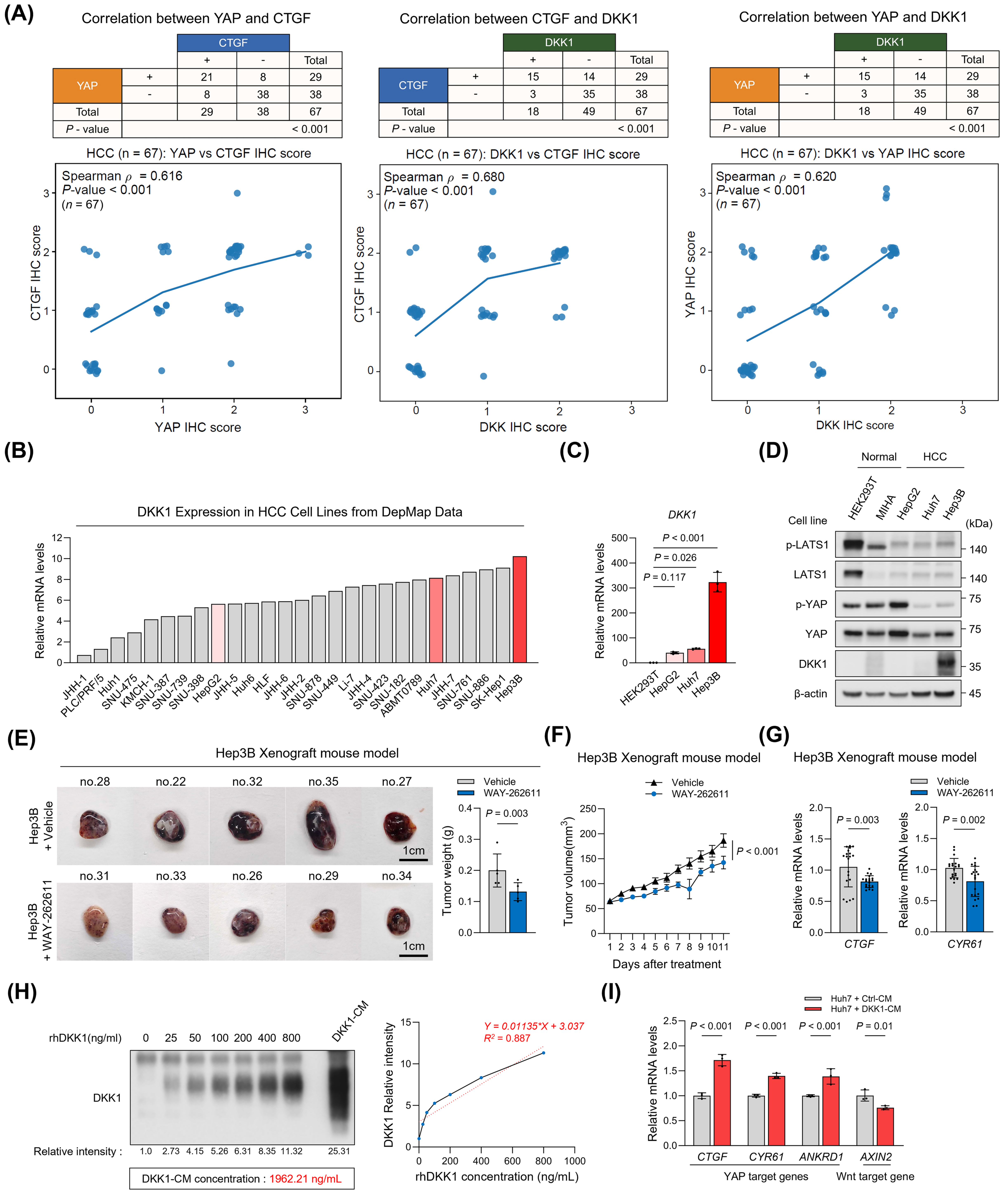
(A)
Correlation between YAP and CTGF
CTGF + - Total
YAP + 21 8 29
- 8 38 38
Total 29 38 67
P - value < 0.001
Correlation between CTGF and DKK1
DKK1 + - Total
CTGF + 15 14 29
- 3 35 38
Total 18 49 67
P - value < 0.001
Correlation between YAP and DKK1
DKK1 + - Total
YAP + 15 14 29
- 3 35 38
Total 18 49 67
P - value < 0.001
HCC (n = 67): YAP vs CTGF IHC score
Spearman ρ = 0.616
P-value < 0.001
(n = 67)
CTGF IHC score
YAP IHC score
HCC (n = 67): DKK1 vs CTGF IHC score
Spearman ρ = 0.680
P-value < 0.001
(n = 67)
CTGF IHC score
DKK IHC score
HCC (n = 67): DKK1 vs YAP IHC score
Spearman ρ = 0.620
P-value < 0.001
(n = 67)
YAP IHC score
DKK IHC score
(B)
DKK1 Expression in HCC Cell Lines from DepMap Data
Relative mRNA levels
JHH-1 PLC/PRF/5 Huh1 SNU-475 KMCH-1 SNU-387 SNU-739 SNU-398 HepG2 JHH-5 Huh6 HLF JHH-6 JHH-2 SNU-878 SNU-449 Li-7 JHH-4 SNU-423 SNU-182 ABMT0789 Huh7 JHH-7 SNU-761 SNU-886 SK-Hep1 Hep3B
(C)
DKK1
Relative mRNA levels
P < 0.001
P = 0.026
P = 0.117
HEK293T HepG2 Huh7 Hep3B
(D)
Normal HCC
Cell line
HEK293T MIHA HepG2 Huh7 Hep3B
(kDa)
p-LATS1 140
LATS1 140
p-YAP 75
YAP 75
DKK1 35
β-actin 45
(E)
Hep3B Xenograft mouse model
no.28 no.22 no.32 no.35 no.27
Hep3B + Vehicle
1cm
no.31 no.33 no.26 no.29 no.34
Hep3B + WAY-262611
1cm
Vehicle
WAY-262611
Tumor weight (g)
P = 0.003
(F)
Hep3B Xenograft mouse model
Vehicle
WAY-262611
Tumor volume(mm³)
P < 0.001
Days after treatment
(G)
Hep3B Xenograft mouse model
Vehicle
WAY-262611
Relative mRNA levels
P = 0.003
CTGF
P = 0.002
CYR61
(H)
rhDKK1(ng/ml) 0 25 50 100 200 400 800
DKK1-CM
DKK1
Relative intensity : 1.0 2.73 4.15 5.26 6.31 8.35 11.32 25.31
DKK1-CM concentration : 1962.21 ng/mL
Y = 0.01135*X + 3.037
R² = 0.887
DKK1 Relative intensity
rhDKK1 concentration (ng/mL)
(I)
Huh7 + Ctrl-CM
Huh7 + DKK1-CM
Relative mRNA levels
P < 0.001 P < 0.001 P < 0.001 P = 0.01
CTGF CYR61 ANKRD1 AXIN2
YAP target genes
Wnt target gene
