# Supplementary material for: DKK1 Suppresses Hippo Signaling via PIP3–OGT–LRP6 O-GlcNAcylation in Hepatocellular Carcinoma
Source: Cancer Commun (Lond). 2026 Apr 27;46:0022. doi: 10.34133/cancomm.0022 (PMC13112786; doi:10.34133/cancomm.0022)
Supplement: Supplementary 1 — Materials and Methods Tables S1 to S5 Figs. S1 to S16 [file cancomm.0022.f1.zip › Supplementary Figure S3.pdf]

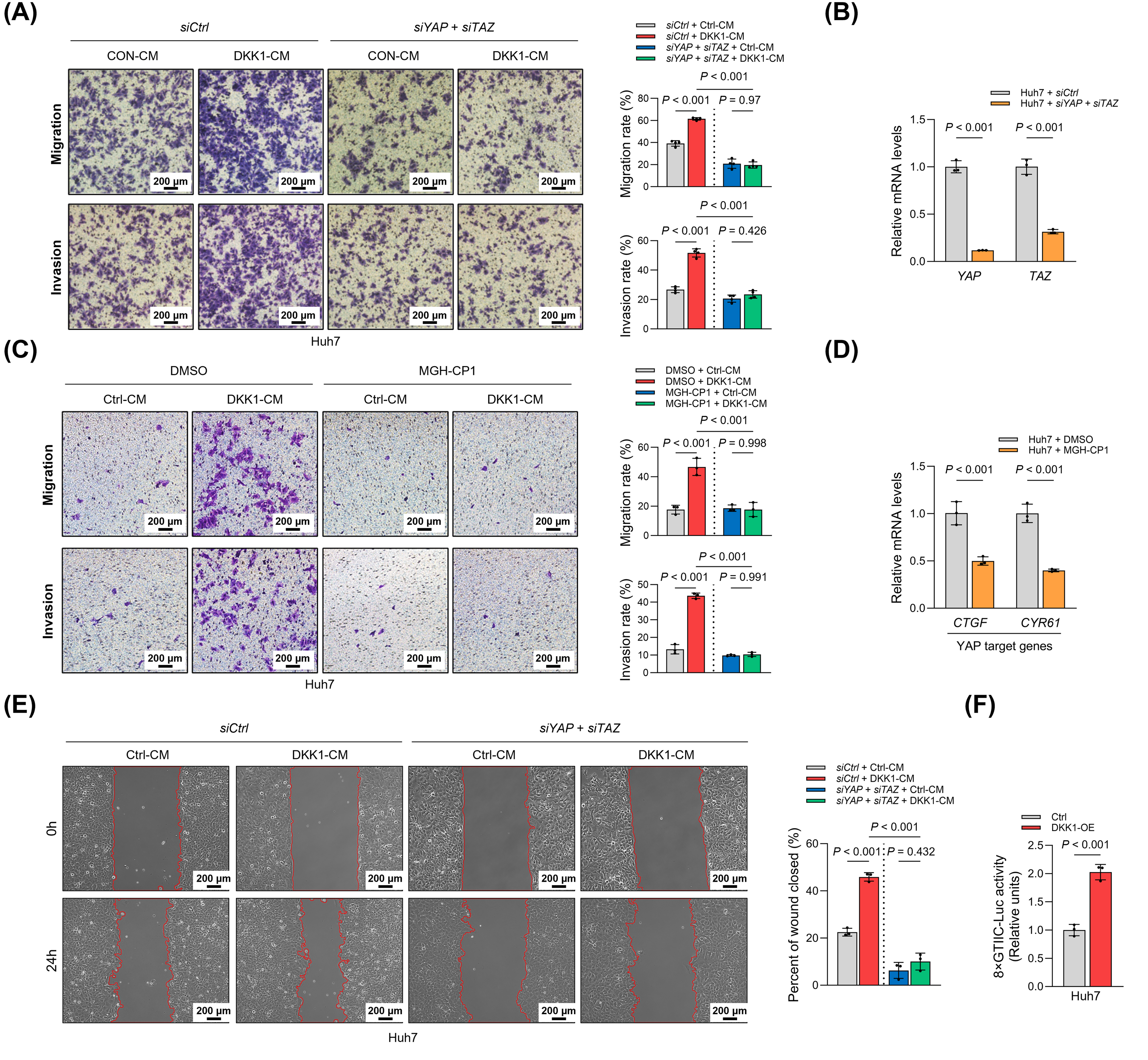

(A)
siCtrl
siYAP + siTAZ
CON-CM
DKK1-CM
CON-CM
DKK1-CM
Migration
Invasion
200 μm
Huh7
siCtrl + Ctrl-CM
siCtrl + DKK1-CM
siYAP + siTAZ + Ctrl-CM
siYAP + siTAZ + DKK1-CM
Migration rate (%)
P < 0.001
P < 0.001
P = 0.97
Invasion rate (%)
P < 0.001
P < 0.001
P = 0.426
(B)
Huh7 + siCtrl
Huh7 + siYAP + siTAZ
Relative mRNA levels
P < 0.001
P < 0.001
YAP
TAZ
(C)
DMSO
MGH-CP1
Ctrl-CM
DKK1-CM
Ctrl-CM
DKK1-CM
Migration
Invasion
200 μm
Huh7
DMSO + Ctrl-CM
DMSO + DKK1-CM
MGH-CP1 + Ctrl-CM
MGH-CP1 + DKK1-CM
Migration rate (%)
P < 0.001
P < 0.001
P = 0.998
Invasion rate (%)
P < 0.001
P < 0.001
P = 0.991
(D)
Huh7 + DMSO
Huh7 + MGH-CP1
Relative mRNA levels
P < 0.001
P < 0.001
CTGF
CYR61
YAP target genes
(E)
siCtrl
siYAP + siTAZ
Ctrl-CM
DKK1-CM
Ctrl-CM
DKK1-CM
0h
24h
200 μm
Huh7
siCtrl + Ctrl-CM
siCtrl + DKK1-CM
siYAP + siTAZ + Ctrl-CM
siYAP + siTAZ + DKK1-CM
Percent of wound closed (%)
P < 0.001
P < 0.001
P = 0.432
(F)
Ctrl
DKK1-OE
P < 0.001
8×GTIIC-Luc activity (Relative units)
Huh7
