# Supplementary material for: DKK1 Suppresses Hippo Signaling via PIP3–OGT–LRP6 O-GlcNAcylation in Hepatocellular Carcinoma
Source: Cancer Commun (Lond). 2026 Apr 27;46:0022. doi: 10.34133/cancomm.0022 (PMC13112786; doi:10.34133/cancomm.0022)
Supplement: Supplementary 1 — Materials and Methods Tables S1 to S5 Figs. S1 to S16 [file cancomm.0022.f1.zip › Supplementary Figure S4.pdf]

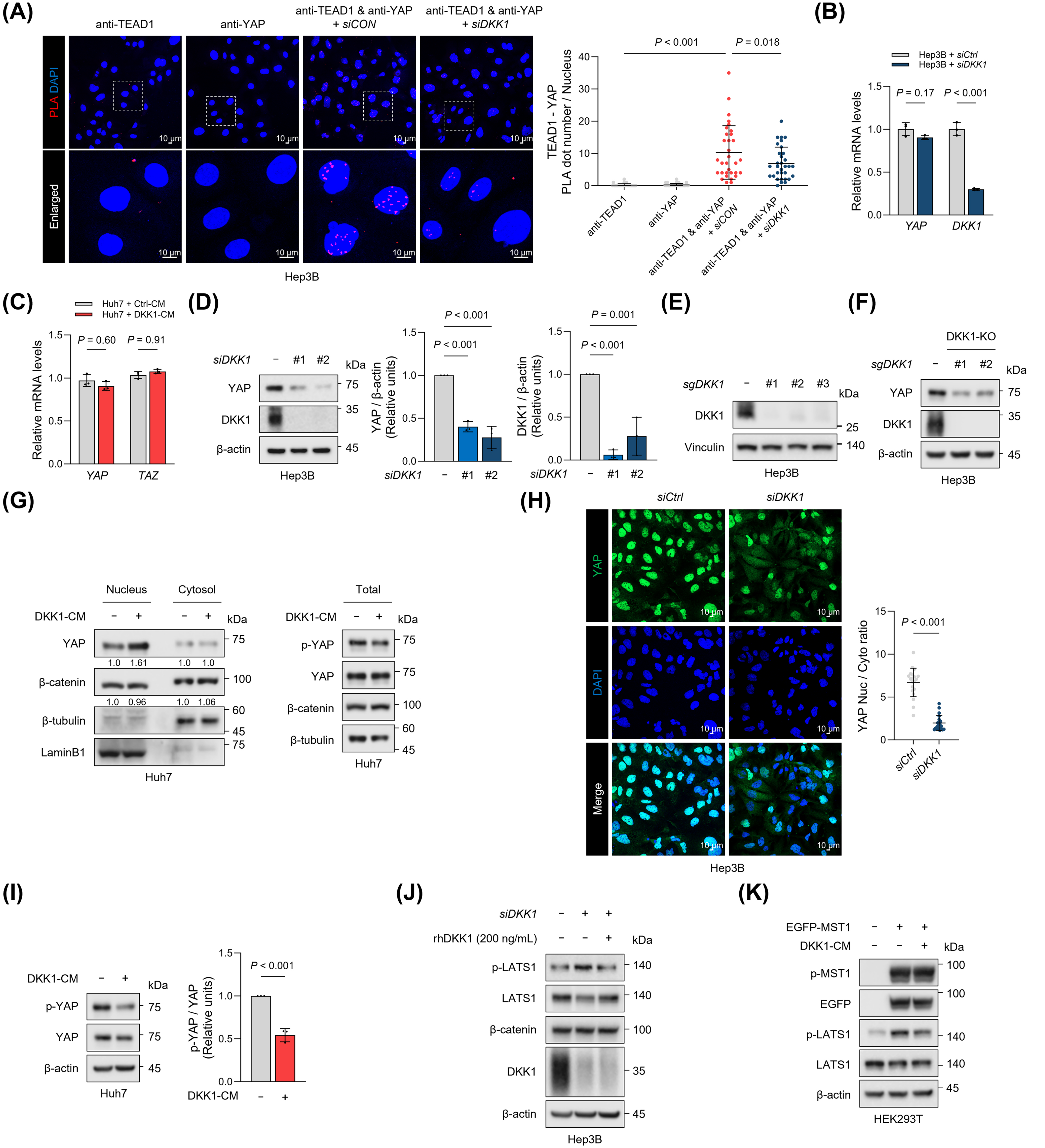
(A)
anti-TEAD1
anti-YAP
anti-TEAD1 & anti-YAP + siCON
anti-TEAD1 & anti-YAP + siDKK1
PLA DAPI
Enlarged
10 μm
Hep3B
TEAD1 - YAP PLA dot number / Nucleus
P < 0.001
P = 0.018
anti-TEAD1
anti-YAP
anti-TEAD1 & anti-YAP + siCON
anti-TEAD1 & anti-YAP + siDKK1
(B)
Hep3B + siCtrl
Hep3B + siDKK1
Relative mRNA levels
P = 0.17
P < 0.001
YAP
DKK1
(C)
Huh7 + Ctrl-CM
Huh7 + DKK1-CM
Relative mRNA levels
P = 0.60
P = 0.91
YAP
TAZ
(D)
siDKK1
– #1 #2
kDa
YAP
DKK1
β-actin
75
35
45
Hep3B
YAP / β-actin (Relative units)
DKK1 / β-actin (Relative units)
P < 0.001
P < 0.001
P = 0.001
P < 0.001
(E)
sgDKK1
– #1 #2 #3
kDa
DKK1
Vinculin
25
140
Hep3B
(F)
DKK1-KO
sgDKK1
– #1 #2
kDa
YAP
DKK1
β-actin
75
35
45
Hep3B
(G)
Nucleus
Cytosol
DKK1-CM
– + – +
kDa
YAP
1.0 1.61 1.0 1.0
β-catenin
1.0 0.96 1.0 1.06
β-tubulin
LaminB1
75
100
60
45
75
Huh7
Total
DKK1-CM
– +
kDa
p-YAP
YAP
β-catenin
β-tubulin
75
75
100
60
45
Huh7
(H)
siCtrl
siDKK1
YAP
DAPI
Merge
10 μm
Hep3B
YAP Nuc / Cyto ratio
P < 0.001
siCtrl
siDKK1
(I)
DKK1-CM
– +
kDa
p-YAP
YAP
β-actin
75
75
45
Huh7
p-YAP / YAP (Relative units)
P < 0.001
DKK1-CM
– +
(J)
siDKK1
– + +
rhDKK1 (200 ng/mL)
– – +
kDa
p-LATS1
LATS1
β-catenin
DKK1
β-actin
140
140
100
35
45
Hep3B
(K)
EGFP-MST1
– + +
DKK1-CM
– – +
kDa
p-MST1
EGFP
p-LATS1
LATS1
β-actin
100
100
140
140
45
HEK293T
