# Supplementary material for: DKK1 Suppresses Hippo Signaling via PIP3–OGT–LRP6 O-GlcNAcylation in Hepatocellular Carcinoma
Source: Cancer Commun (Lond). 2026 Apr 27;46:0022. doi: 10.34133/cancomm.0022 (PMC13112786; doi:10.34133/cancomm.0022)
Supplement: Supplementary 1 — Materials and Methods Tables S1 to S5 Figs. S1 to S16 [file cancomm.0022.f1.zip › Supplementary Figure S5.pdf]

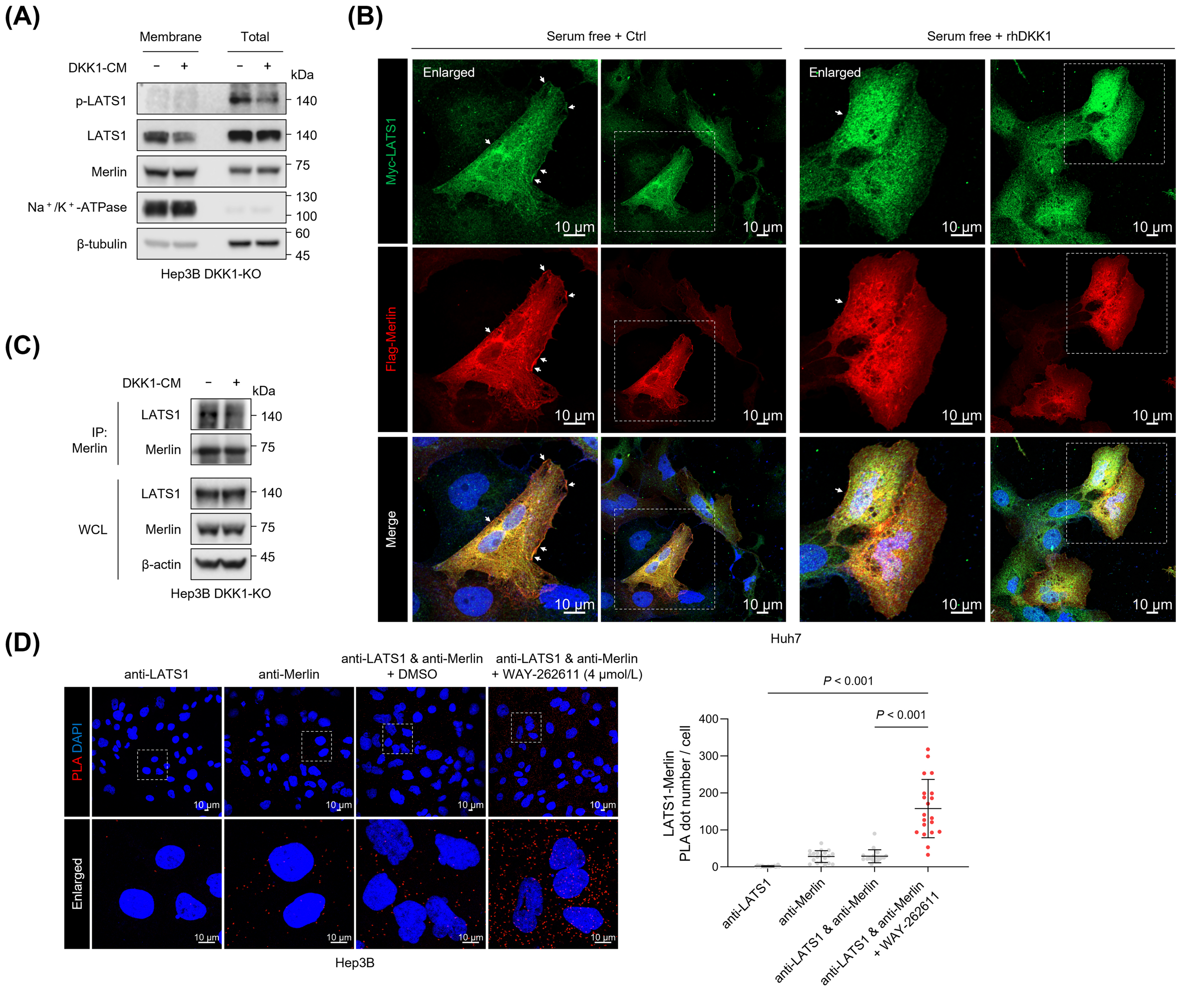

(A)
Membrane
Total
DKK1-CM
– + – +
kDa
p-LATS1
LATS1
Merlin
Na⁺/K⁺-ATPase
β-tubulin
140
140
75
130
100
60
45
Hep3B DKK1-KO
(B)
Serum free + Ctrl
Serum free + rhDKK1
Enlarged
Myc-LATS1
Flag-Merlin
Merge
10 μm
Huh7
(C)
DKK1-CM
– +
kDa
IP: Merlin
LATS1
Merlin
WCL
LATS1
Merlin
β-actin
140
75
140
75
45
Hep3B DKK1-KO
(D)
anti-LATS1
anti-Merlin
anti-LATS1 & anti-Merlin + DMSO
anti-LATS1 & anti-Merlin + WAY-262611 (4 μmol/L)
PLA DAPI
Enlarged
10 μm
Hep3B
P < 0.001
P < 0.001
LATS1-Merlin PLA dot number / cell
400
300
200
100
0
anti-LATS1
anti-Merlin
anti-LATS1 & anti-Merlin
anti-LATS1 & anti-Merlin + WAY-262611
