# Supplementary material for: DKK1 Suppresses Hippo Signaling via PIP3–OGT–LRP6 O-GlcNAcylation in Hepatocellular Carcinoma
Source: Cancer Commun (Lond). 2026 Apr 27;46:0022. doi: 10.34133/cancomm.0022 (PMC13112786; doi:10.34133/cancomm.0022)
Supplement: Supplementary 1 — Materials and Methods Tables S1 to S5 Figs. S1 to S16 [file cancomm.0022.f1.zip › Supplementary Figure S6.pdf]

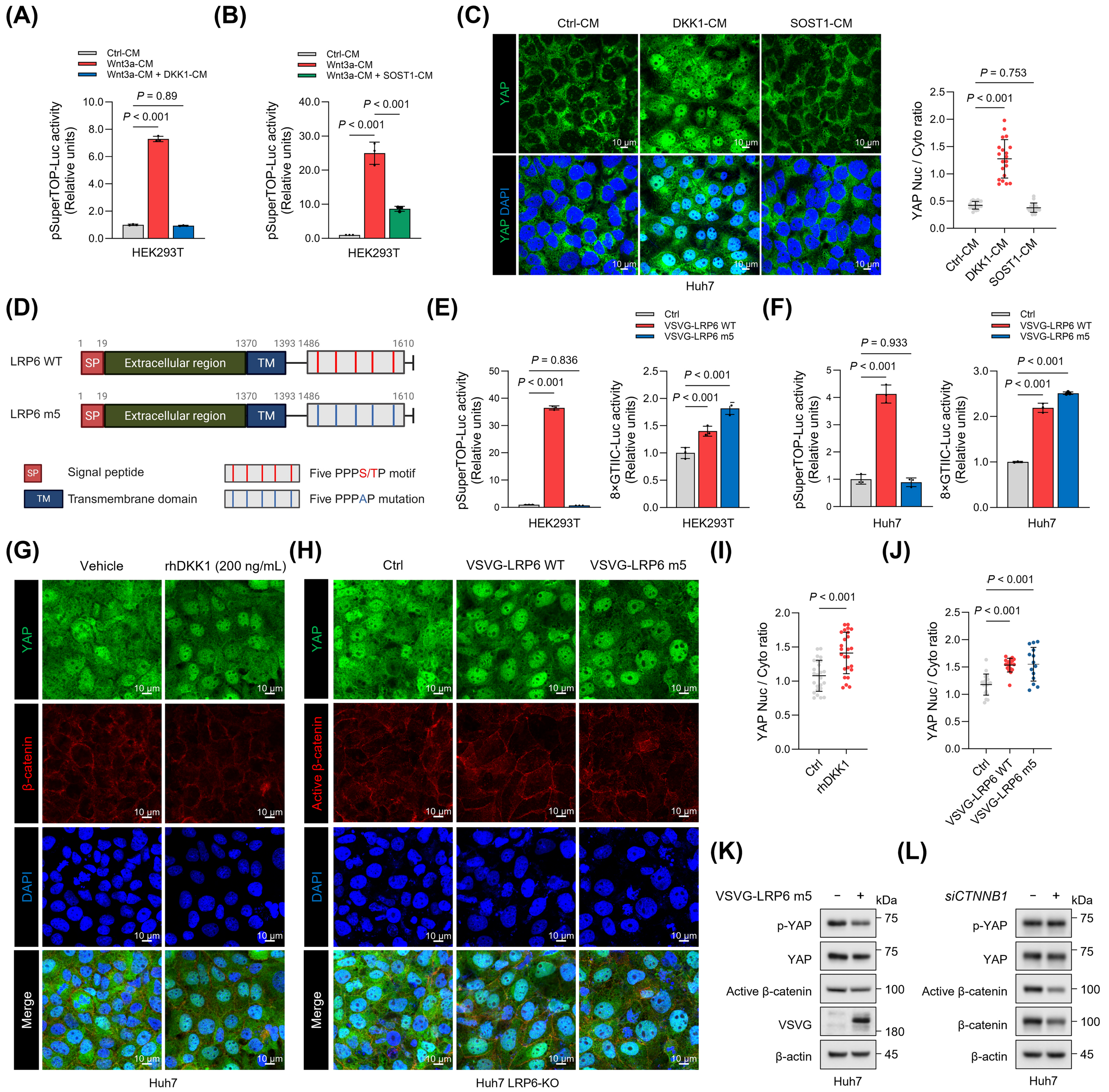
(A)
Ctrl-CM
Wnt3a-CM
Wnt3a-CM + DKK1-CM
pSuperTOP-Luc activity (Relative units)
P = 0.89
P < 0.001
HEK293T
(B)
Ctrl-CM
Wnt3a-CM
Wnt3a-CM + SOST1-CM
P < 0.001
P < 0.001
HEK293T
(C)
Ctrl-CM
DKK1-CM
SOST1-CM
YAP
YAP DAPI
10 μm
Huh7
YAP Nuc / Cyto ratio
P = 0.753
P < 0.001
(D)
LRP6 WT
LRP6 m5
SP
Extracellular region
TM
1 19 1370 1393 1486 1610
Signal peptide
Transmembrane domain
Five PPPS/TP motif
Five PPPAP mutation
(E)
Ctrl
VSVG-LRP6 WT
VSVG-LRP6 m5
pSuperTOP-Luc activity (Relative units)
P = 0.836
P < 0.001
8×GTIIC-Luc activity (Relative units)
P < 0.001
P < 0.001
HEK293T
(F)
P = 0.933
P < 0.001
P < 0.001
P < 0.001
Huh7
(G)
Vehicle
rhDKK1 (200 ng/mL)
YAP
β-catenin
DAPI
Merge
Huh7
(H)
Ctrl
VSVG-LRP6 WT
VSVG-LRP6 m5
Active β-catenin
Huh7 LRP6-KO
(I)
P < 0.001
YAP Nuc / Cyto ratio
Ctrl
rhDKK1
(J)
P < 0.001
P < 0.001
Ctrl
VSVG-LRP6 WT
VSVG-LRP6 m5
(K)
VSVG-LRP6 m5 – +
kDa
p-YAP 75
YAP 75
Active β-catenin 100
VSVG 180
β-actin 45
Huh7
(L)
siCTNNB1 – +
kDa
p-YAP 75
YAP 75
Active β-catenin 100
β-catenin 100
β-actin 45
Huh7
