# Supplementary material for: DKK1 Suppresses Hippo Signaling via PIP3–OGT–LRP6 O-GlcNAcylation in Hepatocellular Carcinoma
Source: Cancer Commun (Lond). 2026 Apr 27;46:0022. doi: 10.34133/cancomm.0022 (PMC13112786; doi:10.34133/cancomm.0022)
Supplement: Supplementary 1 — Materials and Methods Tables S1 to S5 Figs. S1 to S16 [file cancomm.0022.f1.zip › Supplementary Figure S7.pdf]

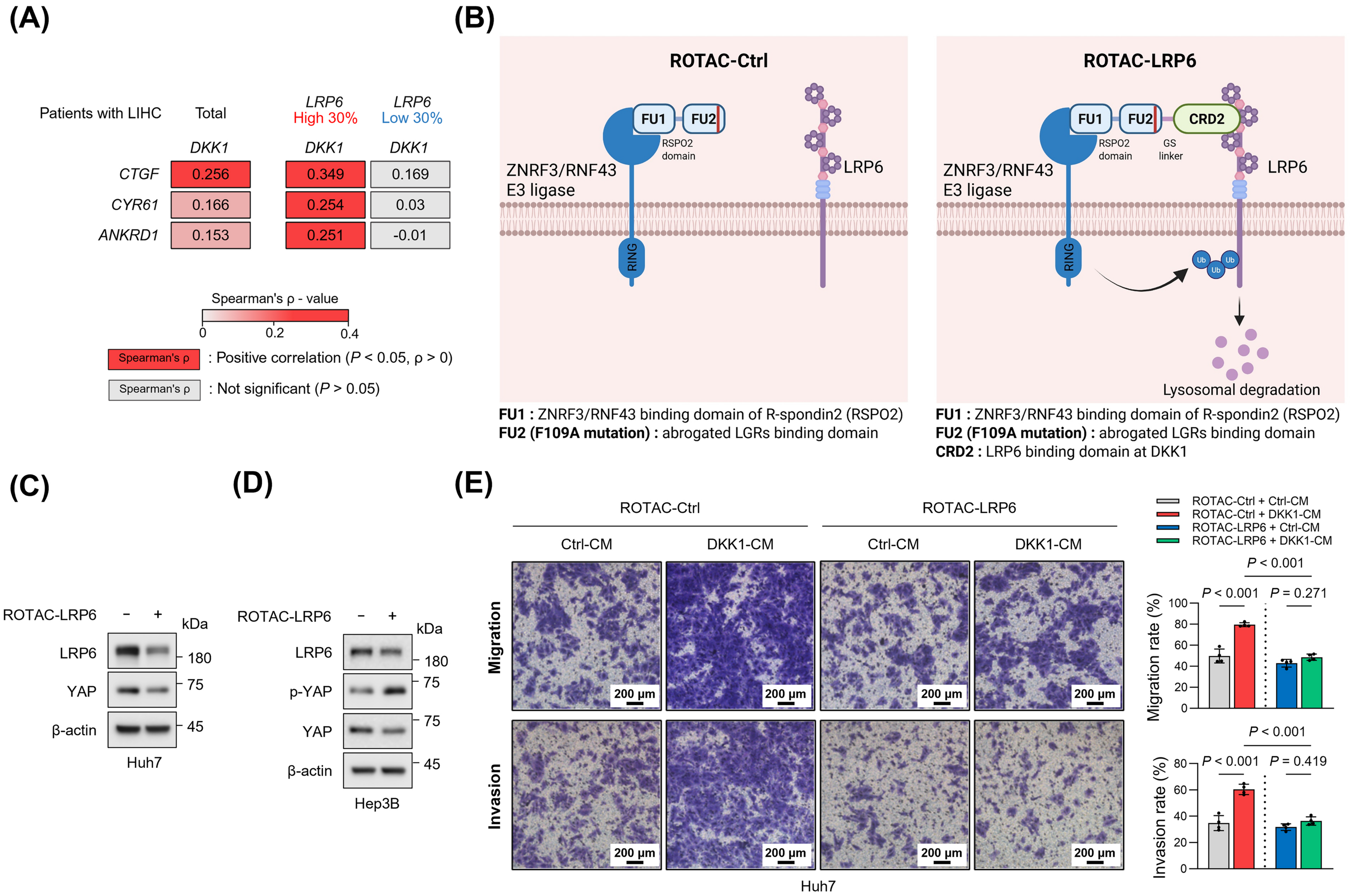

(A)
Patients with LIHC
Total
LRP6 High 30%
LRP6 Low 30%
DKK1
DKK1
DKK1
CTGF 0.256 0.349 0.169
CYR61 0.166 0.254 0.03
ANKRD1 0.153 0.251 -0.01
Spearman's ρ - value
0
0.2
0.4
Spearman's ρ : Positive correlation (P < 0.05, ρ > 0)
Spearman's ρ : Not significant (P > 0.05)
(B)
ROTAC-Ctrl
FU1
FU2
RSPO2 domain
ZNRF3/RNF43 E3 ligase
LRP6
RING
ROTAC-LRP6
CRD2
GS linker
Ub
Lysosomal degradation
FU1 : ZNRF3/RNF43 binding domain of R-spondin2 (RSPO2)
FU2 (F109A mutation) : abrogated LGRs binding domain
FU1 : ZNRF3/RNF43 binding domain of R-spondin2 (RSPO2)
FU2 (F109A mutation) : abrogated LGRs binding domain
CRD2 : LRP6 binding domain at DKK1
(C)
ROTAC-LRP6 - +
kDa
LRP6 180
YAP 75
β-actin 45
Huh7
(D)
ROTAC-LRP6 - +
kDa
LRP6 180
p-YAP 75
YAP 75
β-actin 45
Hep3B
(E)
ROTAC-Ctrl
ROTAC-LRP6
Ctrl-CM
DKK1-CM
Ctrl-CM
DKK1-CM
Migration
Invasion
200 μm
Huh7
ROTAC-Ctrl + Ctrl-CM
ROTAC-Ctrl + DKK1-CM
ROTAC-LRP6 + Ctrl-CM
ROTAC-LRP6 + DKK1-CM
Migration rate (%)
P < 0.001
P < 0.001
P = 0.271
Invasion rate (%)
P < 0.001
P < 0.001
P = 0.419
