# Supplementary material for: DKK1 Suppresses Hippo Signaling via PIP3–OGT–LRP6 O-GlcNAcylation in Hepatocellular Carcinoma
Source: Cancer Commun (Lond). 2026 Apr 27;46:0022. doi: 10.34133/cancomm.0022 (PMC13112786; doi:10.34133/cancomm.0022)
Supplement: Supplementary 1 — Materials and Methods Tables S1 to S5 Figs. S1 to S16 [file cancomm.0022.f1.zip › Supplementary Figure S8.pdf]

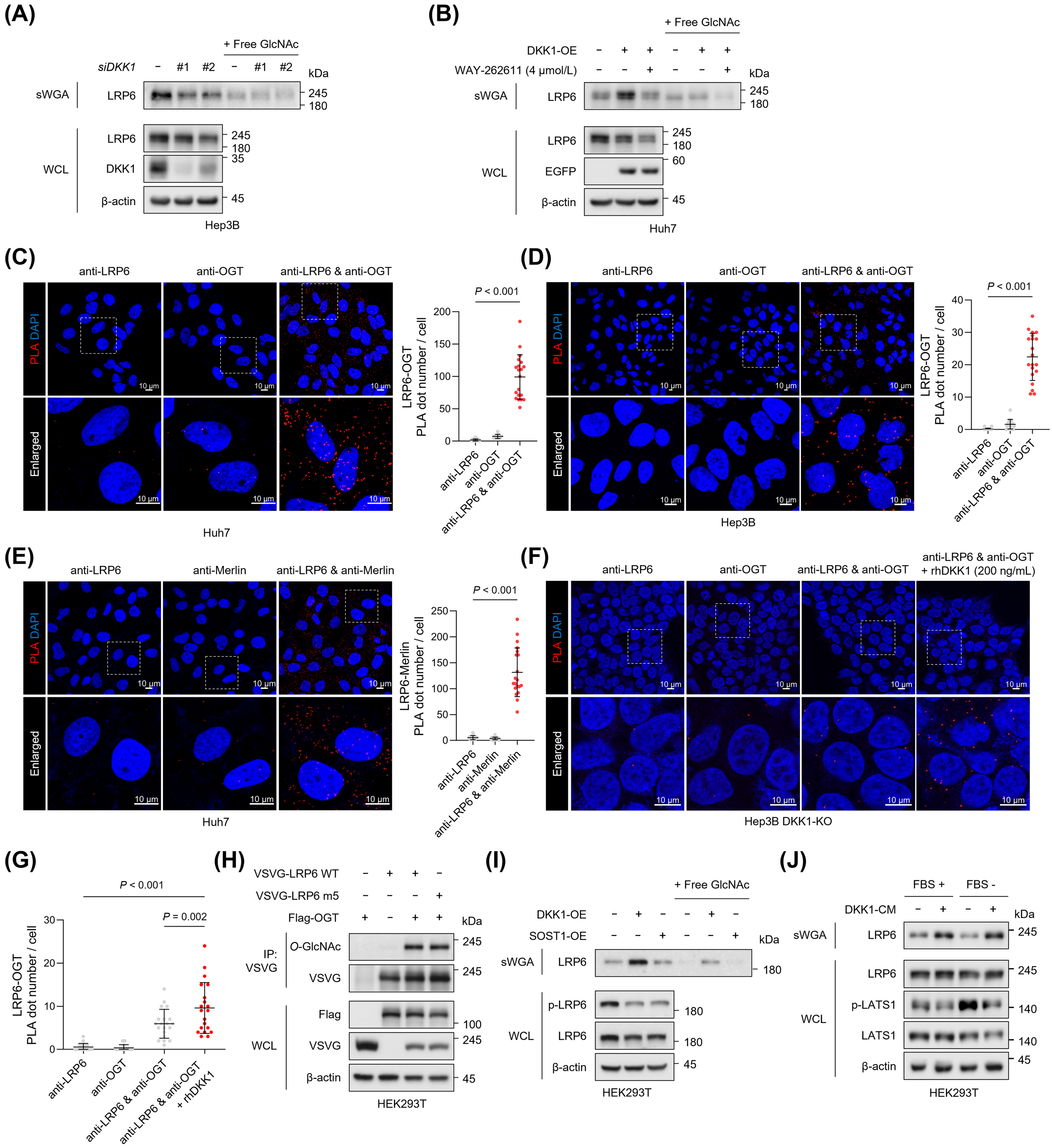
(A)
+ Free GlcNAc
siDKK1 – #1 #2 – #1 #2
kDa
sWGA LRP6 245 180
WCL LRP6 245 180
DKK1 35
β-actin 45
Hep3B
(B)
+ Free GlcNAc
DKK1-OE – + + – + +
WAY-262611 (4 μmol/L) – – + – – +
kDa
sWGA LRP6 245 180
WCL LRP6 245 180
EGFP 60
β-actin 45
Huh7
(C)
anti-LRP6
anti-OGT
anti-LRP6 & anti-OGT
PLA DAPI
Enlarged
10 μm
LRP6-OGT PLA dot number / cell
P < 0.001
Huh7
(D)
anti-LRP6
anti-OGT
anti-LRP6 & anti-OGT
LRP6-OGT PLA dot number / cell
P < 0.001
Hep3B
(E)
anti-LRP6
anti-Merlin
anti-LRP6 & anti-Merlin
LRP6-Merlin PLA dot number / cell
P < 0.001
Huh7
(F)
anti-LRP6
anti-OGT
anti-LRP6 & anti-OGT
anti-LRP6 & anti-OGT + rhDKK1 (200 ng/mL)
Hep3B DKK1-KO
(G)
P < 0.001
P = 0.002
LRP6-OGT PLA dot number / cell
anti-LRP6
anti-OGT
anti-LRP6 & anti-OGT
anti-LRP6 & anti-OGT + rhDKK1
(H)
VSVG-LRP6 WT – + + –
VSVG-LRP6 m5 – – – +
Flag-OGT + – + +
kDa
IP: VSVG O-GlcNAc 245
VSVG 245
WCL Flag 100
VSVG 245
β-actin 45
HEK293T
(I)
+ Free GlcNAc
DKK1-OE – + – – + –
SOST1-OE – – + – – +
kDa
sWGA LRP6 180
WCL p-LRP6 180
LRP6 180
β-actin 45
HEK293T
(J)
FBS + FBS -
DKK1-CM – + – +
kDa
sWGA LRP6 245
WCL LRP6 245
p-LATS1 140
LATS1 140
β-actin 45
HEK293T
