# Supplementary material for: DKK1 Suppresses Hippo Signaling via PIP3–OGT–LRP6 O-GlcNAcylation in Hepatocellular Carcinoma
Source: Cancer Commun (Lond). 2026 Apr 27;46:0022. doi: 10.34133/cancomm.0022 (PMC13112786; doi:10.34133/cancomm.0022)
Supplement: Supplementary 1 — Materials and Methods Tables S1 to S5 Figs. S1 to S16 [file cancomm.0022.f1.zip › Supplementary Figure S9.pdf]

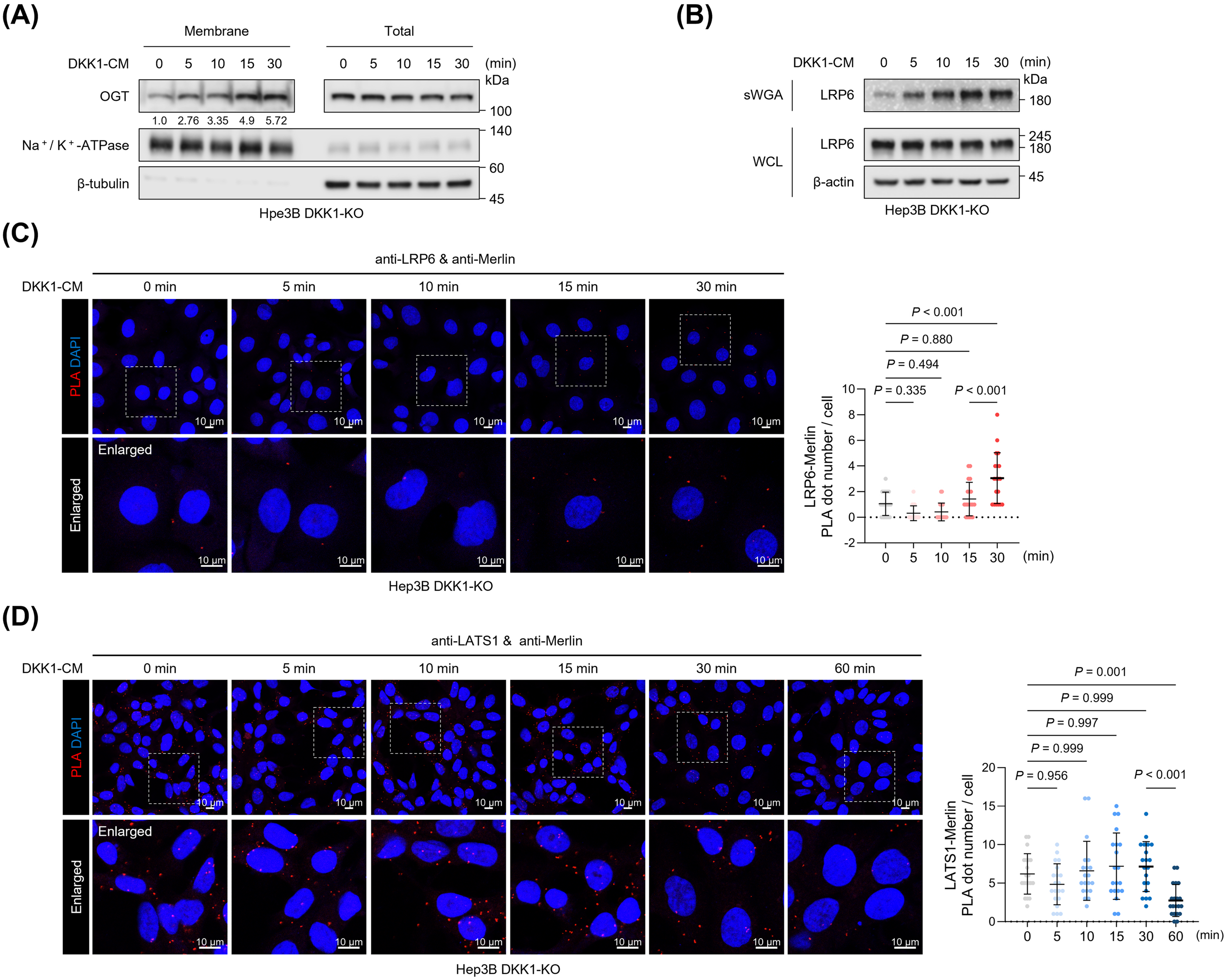
(A)
Membrane
Total
DKK1-CM 0 5 10 15 30
0 5 10 15 30 (min)
kDa
OGT
1.0 2.76 3.35 4.9 5.72
Na⁺/ K⁺-ATPase
β-tubulin
100
140
60
45
Hpe3B DKK1-KO
(B)
DKK1-CM 0 5 10 15 30 (min)
kDa
sWGA
LRP6
180
WCL
LRP6
245
180
β-actin
45
Hep3B DKK1-KO
(C)
anti-LRP6 & anti-Merlin
DKK1-CM
0 min
5 min
10 min
15 min
30 min
PLA DAPI
Enlarged
10 μm
Hep3B DKK1-KO
LRP6-Merlin PLA dot number / cell
P < 0.001
P = 0.880
P = 0.494
P = 0.335
P < 0.001
(min)
(D)
anti-LATS1 & anti-Merlin
DKK1-CM
0 min
5 min
10 min
15 min
30 min
60 min
PLA DAPI
Enlarged
10 μm
Hep3B DKK1-KO
LATS1-Merlin PLA dot number / cell
P = 0.001
P = 0.999
P = 0.997
P = 0.999
P = 0.956
P < 0.001
(min)
